# Bacterial community colonization with minimal alteration of plastics in suboxic sediments from a marine methane seep

**DOI:** 10.1128/aem.02490-25

**Published:** 2026-06-04

**Authors:** Timnit Kefela, Dong Li, Laurie C. Van De Werfhorst, Rebecca M. Reynolds, Rachel Behrens, Yuki Floyd, Jennifer L. Brown, Jen Smith, Michelle A. O’Malley, Patricia A. Holden

**Affiliations:** 1 Bren School of Environmental Science & Management, University of California, Santa Barbara 118683, Santa Barbara, California, USA; 2 Environmental Science and Resource Management Program, California State University, Channel Islands 14703, Camarillo, California, USA; 3 Materials Research Laboratory, University of California, Santa Barbara 8786, Santa Barbara, California, USA; 4 Department of Chemical Engineering, University of California, Santa Barbara 523353, Santa Barbara, California, USA; 5 California NanoSystems Institute, University of California, Santa Barbara 8786, Santa Barbara, California, USA; Michigan State University, East Lansing, Michigan, USA

**Keywords:** EVOH, PET, cellulose acetate, CA, bacterial community, sulfate-reducing bacteria, 16S rRNA gene sequencing

## Abstract

Although marine plastics fragment and sink into suboxic seafloor sediments, little is known regarding microbiological and material outcomes. Here, open glass microcosms containing hydrocarbon-venting marine sediments were amended with either polymer film (three types of ethylene vinyl alcohol or EVOH, a polyethylene terephthalate or PET, and cellulose acetate or CA) or glass control substrates, submerged in a natural seawater flow-through tank, and sampled immediately after deployment (T0) and at approximately 0.5, 1, 3, and 6 months of incubation (T1–T4). Recovered polymer substrates were analyzed for chemical composition, surface morphology, hydrophobicity, crystallinity, and glass transition, melting, and crystallization temperatures. All substrates were colonized, as per extractable DNA. By sequencing genes encoding 16S rRNA, substrate bacterial communities differed substantially from those in seawater and sediments, even at T0, and changed over time, particularly from T3 to T4 (ANOSIM test, all *P* = 0.001). Gammaproteobacteria were initially dominant, and Bacteroidota increased over time. No bacterial genera were relatively abundant on the EVOHs, and only one genus, *Clostridium,* was more abundant on PET. However, several taxa were more abundant on CA, including those from the classes Bacillota and Clostridia, and sulfate-reducing bacteria. According to changes in cellulose backbone spectral abundances of CA polymers, biodegradation was inferred. Evidence for EVOH biodegradation was scant, although abiotic hydrolysis was apparent. Altogether, the polymer substrates were readily colonized, but only CA—according to material alteration and changes to surrounding sediment bacterial communities—appeared to biodegrade, suggesting otherwise slow degradation or broad recalcitrance of the studied plastics in suboxic marine sediments.

**IMPORTANCE:**

Plastic debris entering the ocean is a worldwide problem. Most plastics sink to the seafloor and become buried in marine sediments, yet ecosystem-associated fates of such accumulations are unknown. Additionally, plastics studied for environmental influence and biodegradation in marine ecosystems rarely include laminate polymers such as the ethylene vinyl alcohol copolymers (EVOH) in addition to core polymers such as polyethylene terephthalate (PET). The results of this study indicated CA biodegradation, weak evidence for EVOH biodegradation, and none for PET over the study time frames. Thus, many plastics are likely to persist in suboxic marine sediments.

## INTRODUCTION

Large amounts of plastic debris enter the ocean, estimated at 4.8–12.7 million tons per year (1). Microplastics (plastic particles smaller than 5 mm in size) and nanoplastics (smaller than 1 µm in size) are entering marine food webs, with ingestion by humans via the food chain (2). Biodegradation of plastics is a potential solution for plastic waste management (3, 4), yet plastics persist in the environment, showing slow to low tendencies to fully degrade (5). Meanwhile, bioprospecting and characterization for plastic-degrading microbes have identified isolates that degrade specific polymers (6, 7). Although promising, single isolate activities may incompletely indicate potential biodegradation within the plastisphere, and thus microbial consortia with synergistic dynamics that can result in increased enzyme production and co-metabolism (8) should be understood for pollution management.

Most prior marine studies have focused on biofilms colonizing floating plastic debris, revealing microbial community succession after debris immersion in seawater (9, 10). Plastic debris provides specific ecological niches for microbial colonizers, which can quickly form microbial biofilms within days; these microbial communities attached to plastics are often referred to as the “plastisphere” (10, 11). Floating plastic debris in the ocean is also photo- and thermally oxidized, which may allow for more ready biodegradation (12, 13). However, plastic debris buried in sediments under suboxic and anoxic conditions is not exposed to light and heat, likely decreasing biodegradation relative to surface waters (14). Since most plastic particles in the ocean eventually sink to the seafloor, where they are buried into the seabed under anoxic conditions, with ongoing sedimentation and bioturbation (15) and long-term residence (16), additional research is needed regarding the plastisphere in marine sediments.

Microbes colonize plastics within hours of submergence into sediments (17). Plastisphere succession may occur over months before stabilizing (18), with different microbial communities establishing on plastic debris versus surrounding marine sediments (17–19). Biodegradation of biodegradable and conventional plastics was detected after 98 days of incubation in organic-rich marine sediments, although higher densities of microbes settled on biodegradable plastics than conventional plastics (15). Generally, microorganisms colonizing plastic debris are metabolically active, yet plastic biodegradation in marine sediments is slow (15). This holds even for biodegradable plastics, which are made from natural materials such as cellulose, starch, and bio-based polymers that should be degraded by naturally occurring bacteria, fungi, and algae (20, 21). To date, studies of marine sediment plastispheres and potential biodegradation include the core polymers in packaging laminates, such as poly(ethylene terephthalate) (PET), polypropylene (PP), and polyethylene (PE) (15, 17), but these are simplistic versions of actual plastic debris.

Modern single-use packaging is not a singular polymer but rather a sophisticated layered system of homopolymers and copolymers, with each component serving a distinct functional role, such as surfaces for printing labels, providing structure, and limiting oxygen ingress, with the primary goal to increase the stability of the product (22). Given this complex architecture, effective bioprospecting for microbial consortia capable of degrading single-use packaging requires a systematic approach: each polymer used across these layers must be evaluated to identify which microbes successfully colonize and degrade them. Therefore, to further understand the potential for bacterial colonization and degradation of realistic packaging-associated plastics in marine sediments and to increase the opportunity for bioprospecting, two questions were addressed. First, what is the relative sedimentary plastisphere for common plastic packaging liner material versus other core plastic components? Furthermore, what is the relative apparent biodegradation of the surface versus core polymer material? A range of sediments was not studied; rather, marine sediments within a methane seep were selected, as these contain hydrocarbon-degrading bacterial taxa (23–25) such as *Alcanivorax*, *Marinobacter*, and *Pseudomonas* that have been identified within consortia to biodegrade plastic polymers in the marine aquatic environment (26, 27). Bacterial community assemblies and succession were studied for polymer films of three types of ethylene vinyl alcohol copolymers (EVOH) and polyethylene terephthalate (PET). EVOH, a copolymer of PE and polyvinyl alcohol (PVOH), is widely employed in food packaging due to its excellent gas barrier properties, permeability resistance, and mechanical properties (28, 29), with a global market volume reaching 187,000 tons in 2024 (28). Although EVOH is typically laminated between other plastic layers and is not generally biodegradable, studies have reported biodegradation by specific microorganisms (28) or when blended with materials like thermoplastic starch (30). Furthermore, when compared to other common plastic materials, EVOH is significantly denser than polyolefins and often similar to engineered plastics like PET or nylon (https://www.specialchem.com/plastics/guide/density), indicating that EVOH will eventually also sink to the seafloor and be buried into the seabed (15, 16). PET, a core plastic material that is ubiquitous in textiles and food packaging, comprises 50% of synthetic fiber production (31) and is the third most widely used polymer in packaging worldwide (32). The discovery of PETase in the bacterium *Ideonella sakaiensis* (7) marked a significant breakthrough in understanding PET biodegradation. Degradation of these polymers, however, remains understudied in marine sediments. Cellulose acetate (CA) films and glass coverslips served as control substrates for possible biodegradation and specific colonization, respectively. Microcosms were established using sediments from Brian Seep (Santa Barbara Channel, California), where hydrocarbon gases (primarily methane) vent at the organic-rich sediment seafloor (33, 34). Overall, the results of this study contribute to a better understanding of the marine sediment plastisphere for various relevant plastic polymers and explore the concept of bioprospecting for marine sediment pollution management by specifically examining the methane seep environment.

## MATERIALS AND METHODS

### Experimental setup and operation

To mimic natural environments and evaluate both the bacterial communities colonizing plastic surfaces and their potential biodegradation impact, plastic polymer and glass substrates (Table S1) were buried in hydrocarbon-containing (Table S2) marine sediments *ex situ* within a natural seawater flow-through tank system. Microcosms containing natural sediments were created and randomly placed in an instrumented (Table S3) single gel-coated fiberglass tank (157.48 cm × 83.82 cm; 1.04 m^3^) with continuously flowing unfiltered seawater (average flow rate = 12.3 L/min, Table S4) at the Marine Operations Facility at the University of California, Santa Barbara (UCSB; Fig. S1). Sediments for establishing microcosms were collected from an area of active bubble plumes at Brian Seep (34°N 24.109′; 119°W 49.917′, water depth 10–11 m) in the Santa Barbara Channel on 12/16/2020 (the day of the microcosm establishment) by scientific divers. Seawater was also collected from active bubble plumes at the seep for physical measurements. Details of sample collection and characterization are in the supplemental material. The freshly collected sediment was well mixed in an 11-gallon galvanized steel tub, then distributed into 800 mL PYREX Low Form Griffin Beakers with eight coupons (4 cm × 4 cm) of a single substrate type (plastic polymers or glass controls) buried vertically within. The eight coupons allowed for triplicate samples of adherent bacterial communities to be assessed at each time point; the remaining five coupons were reserved for polymer substrate material characterization analyses (Fig. S2). The flowing unfiltered seawater overtopped the microcosms, and thus the microcosms were fully saturated under the overlying water column, which was in equilibrium with the ambient atmosphere. Six beakers were filled with sediments for each substrate type to allow for five sampling time points. An extra set of five beakers containing sediment only was also prepared to allow for dissolved oxygen measurements. A HOBO Pendant Temperature/Light Data Logger (Onset, Bourne, MA, USA) was mounted inside the tank at the same depth as the sediment level in the beakers to record water temperature and light intensity every 5 min.

The substrate polymers used in this study were oriented polyethylene terephthalate (Futamura Chemical Industries, Oharu-cho Ama-gun, Aichi Pref, Japan), cellulose acetate (Grafix, Maple Heights, OH, USA), and ethylene vinyl alcohol copolymers with differing ethylene (Et) content (Mitsubishi Chemical Corporation, Yokohama, Japan), which were named EVOH1 (Et mol % = 29), EVOH2 (Et mol % = 38), and EVOH3 (Et mol % = 44). Borosilicate glass coverslips were the bacterial colonization control substrates (2.4 cm × 6 cm, Corning Glass, Corning, NY, USA). Additional information regarding substrate (polymer and glass) characteristics is provided in the supplemental material (Table S1).

Prior to deployment, all substrates were surface sterilized using 70% ethanol and/or UV light (254 nm, 10 min). Sterilized substrates (plastic coupons, CA film, and glass coverslips), along with a homogenized sediment sample collected prior to the microcosm setup, served as abiotic material and time 0 controls, respectively. The buried plastic polymer and glass control substrates were recovered for bacterial (all substrates) and material physicochemical (plastic polymers only) characterization analyses at five time points, including immediately after the initial setup (Time 0; T0), 14 days (Time 1; T1), 36 days (Time 2; T2), 104 days (Time 3; T3), and 180 days (Time 4; T4). During each sampling event, one beaker for each substrate type (plastic polymers and glass control) was randomly selected and harvested. From each beaker, three coupons were obtained, gently rinsed three times using filter-sterilized seawater (0.22 µm Supor filters, MicroFunnel Filter Funnels, PALL Co.) to remove any attached sediment particles and archived (−20°C) in sterile 5 mL tubes for DNA extraction. The five plastic polymer substrates retrieved for material physicochemical characterization analyses were rinsed with sterile seawater to remove debris and then shaken and vortexed using Nanopure water (resistivity >18.2 MΩ cm, Thermo Scientific Barnstead, Waltham, MA, USA) at least four times prior to storage in glass Petri plates. The Petri plates were then placed in a glass desiccator with fresh desiccant and left to dry for at least 10 days.

At each sampling time point, seawater from inside the tank was collected into a 4 L sterile polypropylene bottle and 2 to 3 L was filter sterilized to be used for rinsing the coupons. The remaining seawater (1 to 2 L, including *in situ* seawater samples collected prior to microcosm setup; see the supplemental material) was vacuum filtered in the laboratory through sterile 0.22 μm filters (MicroFunnel Filter Funnels, PALL Co.) until the point of refusal or the entire volume was filtered. For each sampling event, a blank was included by filtering approximately 1.5 L of sterile Nanopure water. For each filtration, the exact volume of water filtered was recorded. Filters were stored (−20°C) until DNA extraction. Sediment samples (~0.5 g wet) were collected from the beakers (at the same depth as the coupons) into sterile 2 mL tubes and stored (−20°C) in duplicate until DNA extraction.

During each sampling event, physical parameters of the experimental setup were recorded, including flow rate of seawater entering the tank, dissolved oxygen, pH, and salinity of both the seawater and the sediments in the collected microcosms using an HQ40d multiparameter meter (Hach, Loveland, OH) (Table S3). Additional 4 L seawater samples from Brian Seep were collected at time points T0, two days after T4, and on 9/24/21 for comparison with influent seawater to the fiberglass tank. At the last sampling event (T4), two scraping samples from the side and bottom surfaces of the fiberglass tank were collected for analysis.

### Abiotic control experiment

An abiotic control experiment was performed over 90 days to differentiate potential biodegradation from abiotic (e.g., chemically driven) polymer substrate degradation. Sediments were homogenized in a stainless-steel tray at room temperature, then covered with aluminum foil and autoclaved four times (121°C for 20 min) with 3 days storage at 23°C in between, for fully degrading microbes and associated DNA and RNA (35). Sterilized sediments were subsampled and used to create a 1 mL slurry with sterile seawater (filtered through a 0.22 µm Supor filter; MicroFunnel Filter Funnels, PALL Co.) under a sterile biohood, then streaked on Luria broth (LB) agar plates in triplicate and incubated (35°C, 10 days), showing no microbial growth in comparison to the unsterilized control. In a sterile biohood, sterile sediment and seawater from Brian Seep were added to autoclaved 24 oz. wide-mouth borosilicate glass jars, which were covered with PYREX glass Petri plates. Four sterile coupons (each 4 cm by 4 cm) of each polymer type (i.e., EVOH1, EVOH2, EVOH3, PET, and CA) were buried vertically into the sediments, similarly to the main (live) microcosm experiment. The microcosms were placed in a temperature and light-controlled room mimicking the physical conditions of the live microcosm experiment (see the supplemental material). A HOBO Pendant Temperature/Light Data Logger (Onset, Bourne, MA, USA) was placed in a separate sediment- and seawater-filled jar to record temperature and light intensity at the sediment surface every 5 min. The data were compared to the main (live) microcosm experimental data to establish that the physical abiotic system was representative of the biotic system conditions (Fig. S3 and S4).

### Physicochemical analyses of the polymer substrates

Physicochemical characterization of the cleaned harvested polymer substrates was of the surfaces and the bulk material, the latter including inherent polymer properties such as glass transition temperature and melting temperature, which would signify extensive depolymerization and modification of the polymer (see the supplemental material). The measures were applicable for the polymers, including CA, and not the glass controls, which were used for bacterial colonization assessments.

To visualize and quantify whether there was any degradation occurring on the surface of the polymer substrates, micrographs were acquired using a Nova Nano 650 scanning electron microscope (SEM) at the California NanoSystems Institute (CNSI) at UCSB. The substrates were sputter-coated with a 10 nm layer of gold palladium (Anatech USA Hummer Sputtering System), prior to being mounted on standard aluminum SEM stubs and imaged in high vacuum mode using an Everhard-Thornley detector (ETD) with an accelerating voltage of 5.00 kV at 2,600× magnification. Ten micrographs were captured per substrate type and three conditions (i.e., pristine, T4 biotic at 180 days, 90 days abiotic). The subsequent image analyses followed approaches described previously (36). In brief, the SEM micrographs were analyzed in ArcGIS Pro (version 2.4.0; ESRI, Redlands, CA) using the variety statistic in the Neighborhood tool. The pixel kernel size, which establishes the scale at which the variety is assessed, was dictated by the images—specifically the magnification (2,600×) and the pixel density—while taking into consideration the pixel length in each micrograph that corresponds to 0.2 µm, a value selected to be smaller than the average bacteria size (i.e., 1 µm) to visualize potentially bacterially-mediated topographical changes.

To further determine any changes in the surface chemistry of the polymer substrates, attenuated total reflectance Fourier transformed infrared spectroscopy (ATR-FTIR) was performed using a Thermo Nicolet iS10 FTIR Spectrometer with a Smart Diamond ATR accessory at the TEMPO facility in the Materials Research Laboratory at UCSB. The FTIR spectra were collected in the region of 4,500–400 cm^−1^ with 64 scans at a resolution of 2 cm^−1^. ATR-FTIR was performed for pristine, abiotic, and both biotic (T3 and T4) polymer samples. Five independent spectra were acquired for each polymer substrate: one at each of the four corners, and one at a center point.

Contact angle goniometry was performed to assess changes in hydrophobicity of the polymer substrate surfaces for the pristine, abiotic, and T4 polymer substrates (see the supplemental material). An automated Rame-Hart 290-U1 Goniometer with DROPimage (version 3.19.12.0) was used to measure sessile liquid drop (SLD) contact angles deposited on the four corners and center of the substrates’ surfaces (which were cut to 1 cm × 1 cm sub-samples) mounted and adhered flat on a glass microscope slide. SLD contact angle measurements were performed by depositing a 3 µL drop of Milli-Q water onto the dry film’s surface. The distance between the substrate surface and the dispensing pipette tip was fixed, thereby avoiding the gravitational effects of liquid drop deposition. The digital U1 series camera was used to capture images of the SLD. These images were then analyzed using the DROPimage software, which measured the apparent static contact angles on the left and right sides of each SLD (37, 38).

Differential scanning calorimetry (DSC) was conducted on the pristine, abiotic, and T4 biotic polymer samples (see the supplemental material) to determine the glass transition temperature (*T*_g_), melting temperature (*T*_m_), and the crystallization temperature (*T*_c_) using a DSC 2500 (TA Instruments, Newcastle, DE, USA) for a 5 mg sample with a heating and cooling rate of 10°C/min in nitrogen atmosphere. The cycle program for all EVOHs entailed heating from −10°C to 225°C, followed by cooling to −10°C, repeated thrice. The cycle program for CA was heating from −160°C to 225°C, followed by cooling to −160°C, repeated thrice. Lastly, the cycle program for PET entailed heating from −20°C to 300°C, followed by cooling to −20°C, repeated thrice. Final data were obtained from two full cycles, which were identical. The inflection points of the DSC traces were used for the determination of the *T*_g_. The *T*_m_ and *T*_c_ data were obtained from the peak maximum temperature of the integrated melting and crystallization peaks, respectively, on TRIOS (version 5.1.1; TA Instruments, Newcastle, DE, USA). The degree of crystallinity (*X*_*c*_; %) was assessed by DSC for all polymer substrates across the different conditions by using the following calculation:


$$X_{c}\left(\%\right)=\frac{\Delta H_{m}}{\Delta H_{m^{{}^{\circ }}}}\times 100$$

The enthalpy of melting (Δ*H*_*m*_) was determined by integrating the area under the melting curves and peaks on TRIOS (version 5.1.1; TA Instruments, Newcastle, DE, USA), respectively. The enthalpy of melting values for a 100% crystalline polymer (Δ*H*_*m*_°) for each studied polymer were obtained from the published literature, specifically 217.8 J/g for EVOH (39) and 140.1 J/g for PET (40). The change in crystallinity could not be determined for CA, as it is likely that the endothermic peak is not a melting peak, but instead an induced glass chemical transition, as supported by the lack of endothermic peaks in subsequent scans (41).

Sample preparation for all these analyses occurred under a laminar flow hood or near a benchtop with an air filter to limit airborne particulate contamination of the polymer substrate surfaces. Substrates were stored pre-analysis in either acid-washed (10% HCl) glass Petri plates or in trace-clean borosilicate glass vials.

### DNA extraction, PCR, and sequencing

DNA from the polymer substrates, glass control slides, and filters was extracted using the DNeasy PowerWater Kit (Qiagen, Carol Stream, IL) following the manufacturer’s protocol. DNA from the sediment samples was extracted using the DNeasy PowerSoil Kit or the DNeasy PowerSoil Pro Kit (Qiagen, Carol Stream, IL). Each sediment sample duplicate was extracted separately and combined into a single spin filter during the extraction process. An extraction blank without any filter was included in each batch of DNA extractions for all sample types (water, coupon, and sediment). DNA concentration was quantified using the Quant-iT dsDNA Broad-Range Assay Kit (Invitrogen, Carlsbad, CA) on a Cytation3 Cell Imaging Multi-Mode Reader (BioTek Instruments, Inc., Winooski, VT).

The 27F forward primer (5′-AGAGTTTGATCCTGGCTCAG-3′) and the 534R reverse primer (5′-ATTACCGCGGCTGCTGG-3′), which target the V1-V3 region of genes encoding 16S rRNA, were used for PCR amplification (details in the supplemental material). Mock communities were not included in the sequencing analysis. Filter blanks, extraction blanks, and no-template controls were used as negative controls. Amplification products were purified with Agencourt AMPure XP beads (Beckman Coulter Genomics) and quality checked using the Agilent TapeStation. The Nextera XT Index Kit was used to attach dual indices and Illumina sequencing adapters to the amplicons. After purification with AMPure XP beads and quality checking using TapeStation, amplicons were normalized and pooled prior to paired-end sequencing on the Illumina MiSeq platform with a MiSeq v3 600-cycle kit (2 by 300 bp) in the California NanoSystems Institute (CNSI), UCSB.

### Data analyses

Illumina sequencing data were processed using the Quantitative Insights Into Microbial Ecology (QIIME v1.9.1) pipeline with default settings (42). Paired-end reads were assembled with a minimum 100 base pair (bp) overlap. The minimum sequence length was set to 460 bp. After quality filtering, 20,751,662 sequences were obtained for all samples. To compare samples of varying sample size, the number of sequences per sample was rarified to an even depth of 22,836 sequences. Sequences were grouped into operational taxonomic units (OTUs) at 97% sequence similarity. Representative sequences for each OTU were picked, and taxonomic data were assigned using the Greengenes 13_8 aligned reference database (43). Possible chimeric sequences were checked with the ChimeraSlayer wrapper in QIIME.

The relative abundance of bacterial species in each sample was transformed (log(x + 1)) for normalization, and then similarity matrices for all samples were calculated using Bray–Curtis distance. Principal coordinate analysis (PCoA) based on sample similarity matrices was performed using PRIMER 6 (44). To explore the presence of potential biodegrading bacteria and the impacts of plastics on surrounding sediment communities, bacterial genera significantly more abundant in seawater, sediments, and on each substrate (plastic and glass) were determined using the LEfSe (the linear discriminant analysis effect size) algorithm (45) as well as the DESeq2 method within QIIME. The results of the LEfSe and DESeq2 methods for each type of sample were combined and ranked based on the relative abundance of identified bacterial genera. The top genera were selected with their relative abundances displayed in heatmaps. Core bacterial genera on plastic and glass substrates were defined as present in more than 97% of substrate samples. Bacterial genera enriched in seawater, sediments, and each coupon (plastic and glass) were determined by calculating the Spearman’s correlation coefficient between the relative abundance of each bacterial genus and incubation days using JMP 10 (SAS, Cary, NC). Bacterial species in each sample of this study were further compared to the 875 species of microorganisms listed in PlasticDB that were reported to have plastic-degrading capabilities to identify the potential biodegraders of plastics in this study (6). The Chao1 Richness was calculated in QIIME.

The origin of bacterial colonizers on substrates was predicted by source proportion analysis using SourceTracker 1.0 with default parameters (46). To analyze the origins of bacteria on polymer and glass substrates, bacterial communities identified in the seawater and sediments were designated as sources, and the bacterial communities on substrates were designated as sinks. Average source proportions were obtained by running SourceTracker in triplicate. Heatmaps were generated using Heatmapper (47).

Additional statistical analyses, such as the Mann–Whitney test and analysis of similarities (ANOSIM) test, were performed using JMP 10 (SAS, Cary, NC) or PRIMER 6. PERMDISP (permutation analysis of dispersion) was performed for the significance test of ANOSIM to confirm the homogeneity of dispersions (variances) by non-significant *P*-values.

Statistical analyses on most polymer characterization data (contact angle, mean variety, and DSC results) were performed by one-way analysis of variance (ANOVA) using OriginPro 2023 software (48). Pairwise comparisons of average values for these data were evaluated using Tukey’s honestly significant difference (HSD) post hoc test at a confidence interval (CI) of 95%, that is, *P* < 0.05. The ATR-FTIR data for each polymer coupon were analyzed by first calculating the mean and standard error across the five independent absorbance values for each characteristic wavenumber. The differences across samples (i.e., pristine, abiotic, and biotic at 104 and 180 days), when evaluating within polymer type and wavenumber, were calculated by the Wilcoxon signed-rank test in JMP 10 (SAS, Cary, NC). To interpret ATR-FTIR absorbance changes within wavenumber as from either abiotic or biotic (e.g., biodegradation) factors, single variate regressions were calculated by using the mean values of each wavenumber (Microsoft Excel) and, when the slope of change between the pristine and biotic data was equal to or less than that between pristine and abiotic data, changes were deduced as only attributable to abiotic factors; possible biotic factors were inferred as contributing when the absorbance change slope between pristine and 104 d biotic samples exceeded that between pristine and abiotic samples.

## RESULTS

### Increasing surface heterogeneity of polymers during incubation

Polymer substrate analyses included acquiring SEM micrographs (Fig. S5) and evaluating the images of cleaned coupons for heterogeneity due to potential biofilm-mediated surface degradation, such as pitting, notching, and cracks (49). Substrate surface heterogeneity was defined by determining the mean variety value for each polymer type from T4 (180 d) of the biotic experiment and the 90-day abiotic experiment in comparison to the pristine films. Owing to the different incubation times for abiotic versus biotic samples (90 d vs. 180 d, respectively), biodegradation could not be inferred from these results (see the supplemental material).

For EVOH1–3, polymer surface heterogeneity, as indicated by mean variety, statistically differed across all conditions (*P* < 0.05; Table 1). For EVOH1, pairwise comparisons of the surfaces between the pristine, 90 d abiotic, and T4 biotic conditions were significantly different, that is, more heterogeneous, between the pristine polymers and the 90 d abiotic and T4 biotic treatments (all *P* < 0.05) but showing no difference in the comparisons between the 90 d abiotic and T4 biotic treatments (Table 1; Fig. S5). Similarly, EVOH2 and EVOH3 pairwise comparisons showed significant differences between the pristine polymers, 90 d abiotic, and T4 biotic, including differences between the abiotic and biotic conditions (*P* < 0.05; Table 1; Fig. S5). The increased heterogeneity of the abiotic exposure EVOH films in comparison to the pristine is likely due to the plasticization of EVOH by water (50). The ethylene content correlated with the surface heterogeneity on the EVOHs incubated in the abiotic treatment group, whereby EVOH1 showed more heterogeneity in comparison to both EVOH2 and EVOH3 (Table 1). However, in the T4 biotic condition, there were no significant differences across all three EVOHs, despite the difference in ethylene content.

**TABLE 1:** Characterization of the polymer substrates toward quantifying the time course of surface hydrophobicity by contact angle measurement, surface heterogeneity and surface roughness by mean variety value of SEM images (Fig. S5), melting and crystallization temperatures, and degree of crystallinity as a percentage^*a*^

Plastic	Contact angle (°)	Mean variety value	*T*_m_ (°C)	*T*_c_ (°C)	*X*_*c*_ (%)
EVOH1					
Pristine	89.07 ± 2.99^a^	11.42 ± 0.38^a^	186.28 ± 0.40^a^	162.78 ± 5.06^a^	29.9 ± 1.75^a^
90 d (abiotic)	82.14 ± 11.08^b^	33.25 ± 6.23^b^	186.43 ± 0.24^a^	161.70 ± 0.96^a^	28.62 ± 1.18^a^
180 d (biotic)	75.52 ± 14.57^c^	29.52 ± 7.21^b^	185.52 ± 0.96^a^	162.08 ± 2.05^a^	27.52 ± 1.86^a^
EVOH2					
Pristine	82.36 ± 10.15^a^	9.55 ± 0.98^a^	173.44 ± 0.58^a^	143.34 ± 8.20^a^	22.67 ± 2.92^a^
90 d (abiotic)	67.52 ± 9.29^b^	21.79 ± 0.36^b^	173.27 ± 0.59^a^	133.97 ± 0.23^a^	21.60 ± 1.89^a^
180 d (biotic)	68.36 ± 6.69^b^	27.98 ± 2.74^c^	172.75 ± 0.63^a^	150.39 ± 1.22^a^	24.03 ± 1.11^a^
EVOH3					
Pristine	79.68 ± 3.37^a^	8.02 ± 2.84^a^	164.71 ± 1.48^a^	143.67 ± 1.44^a^	25.72 ± 4.93^a^
90 d (abiotic)	70.73 ± 5.16^b^	20.92 ± 0.45^b^	164.59 ± 0.78^a^	144.43 ± 2.37^a^	25.71 ± 1.47^a^
180 d (biotic)	62.51 ± 4.08^c^	27.47 ± 3.48^c^	164.64 ± 0.27^a^	143.62 ± 0.83^a^	25.18 ± 2.35^a^
PET					
Pristine	81.11 ± 2.62^a^	10.28 ± 1.17^a^	253.13 ± 0.55^a^	195.68 ± 0.93^a^	30.94 ± 2.40^a^
90 d (abiotic)	83.87 ± 1.22^a^	11.04 ± 0.26^a^	252.33 ± 0.07^a^	205.23 ± 4.62^a^	29.80 ± 11.89^a^
180 d (biotic)	81.18 ± 4.70^b^	13.23 ± 1.43^b^	252.14 ± 2.49^a^	203.29 ± 12.26^a^	29.88 ± 3.07^a^
CA					
Pristine	57.55 ± 3.33^a^	10.74 ± 0.21^a^	nd	nd	nd
90 d (abiotic)	59.18 ± 1.77^ab^	9.17 ± 1.63^a^	nd	nd	nd
180 d (biotic)	59.38 ± 5.59^b^	12.63 ± 2.34^b^	nd	nd	nd

^*a*^ Statistical comparison results by column denote a *P* value of < 0.05 if they have differing letters (one-way ANOVA with Tukey’s post hoc tests). *T*_m_, melting temperature; *T*_c_, crystallization temperature; % *X*_*c*_, percent crystallinity; nd, not determined.

Similarly to the EVOH polymers, the T4 biotic surfaces (Table 1) of the CA and PET films were more heterogeneous (*P* < 0.05) than for the pristine and abiotic conditions. There was no difference in heterogeneity between the pristine and abiotic CA and PET films, respectively, suggesting that the difference in mean variety value is likely increased surface roughness because of surface erosion, possibly by biofilm-mediated pitting and tunneling (Fig. S5). However, because the incubation time for the biotic samples was double that of the abiotic samples (i.e., 180 d vs 90 d), it was not possible to definitively explain the differences as being from biotic processes.

### Different surface hydrophobicity changes among polymers

The contact angle decreased for both the 90 d abiotic and T4 biotic samples due to increased surface roughness and the pristine polymers having an apparent contact angle of <90° (Table 1). Pairwise comparisons showed significant differences between the pristine, abiotic, and biotic treatments (*P* < 0.05) for EVOH1 and EVOH3. Meanwhile, pairwise comparisons for EVOH2 were significantly different between the pristine and abiotic treatments (*P* < 0.05) and the pristine and biotic treatments (*P* < 0.05). There was no difference between the abiotic and biotic conditions (*P* > 0.05) for EVOH2.

The contact angle of the T4 biotic PET (Table 1) increased and significantly differed (*P* < 0.05) from both the abiotic and pristine conditions. Similarly, the contact angle of the T4 biotic CA increased and significantly differed (*P* < 0.05) from the pristine. There was no difference for CA in the contact angle among the pristine, abiotic, and biotic conditions.

### Surface chemistry suggested minimal degradation of all polymers except CA

ATR-FTIR analysis provides insight into the presence, absence, and changes in concentration of functional groups and the chemical structure of a polymer, based on the absorbance values exhibited for characteristic wavenumbers (51). For ATR-FTIR data analysis, spectra of the abiotic (90 d) samples were compared to those of the T3 biotic samples (104 d), in addition to those of the T4 (180 d) biotic samples, to assign surface chemistry changes as from either abiotic, biotic, or combined influences.

**Fig 1:**
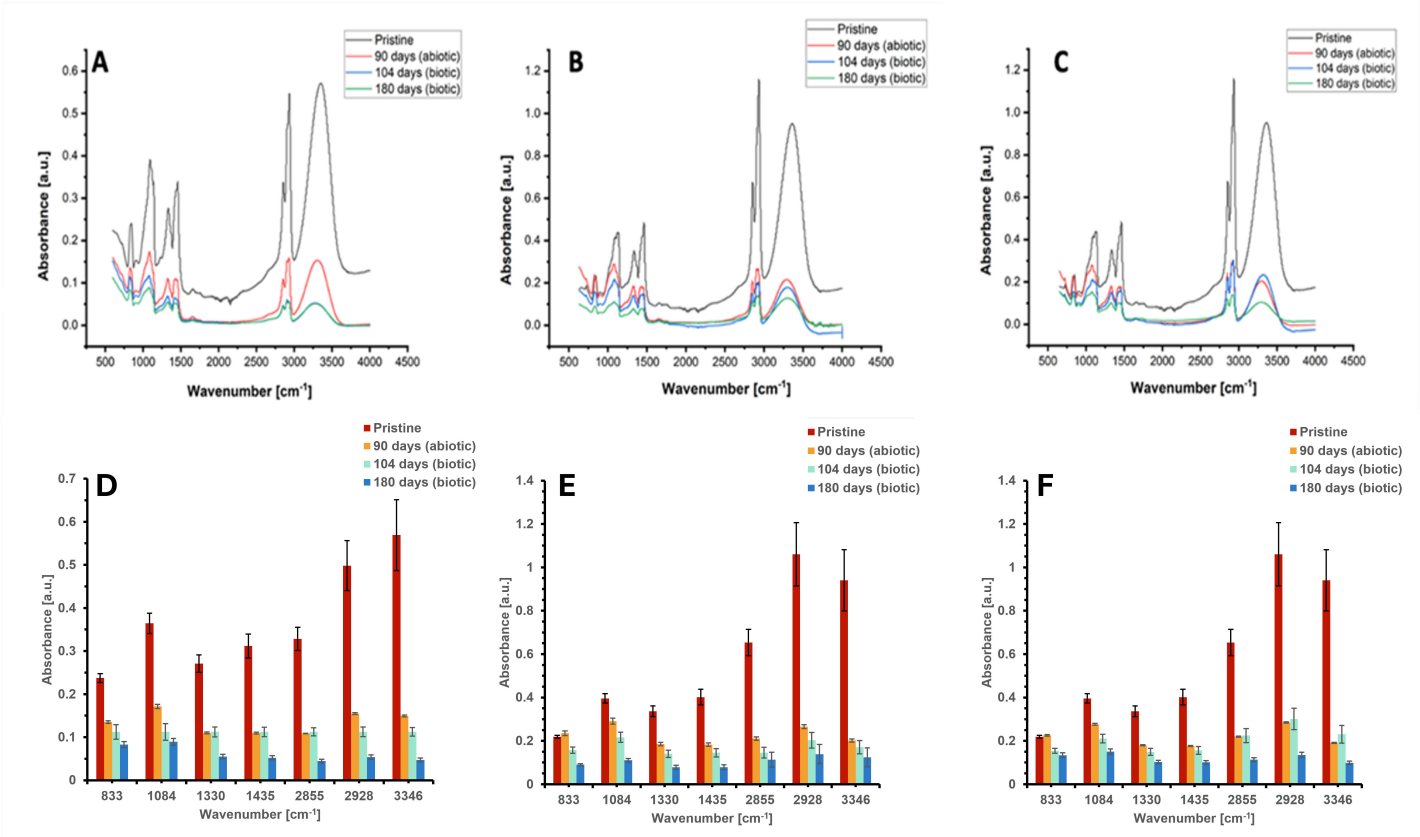
ATR-FTIR spectra (**A–C**) and average absorbances of the representative peaks (**D–F**) of the polymer substrate surfaces across all the relevant conditions in this study (A and D, EVOH1; B and E, EVOH2; C and F, EVOH3). Error bars are relative standard deviation.

The representative peaks for EVOH were 833, 1,084, 1,330, 1,435, 2,855, 2,928, and 3,346 cm^−1^ (Fig. 1A through F), which corresponded to the skeletal vibration, ether (C-O stretching) bonds, alcohol deformation (O-H) bonds (1,330 and 1,435 cm^−1^), alkane groups (C-H symmetric and antisymmetric stretching, 2,855 and 2,928 cm^−1^, respectively), and the hydrogen-bonded hydroxyl (O-H stretching) groups (52, 53), respectively (Table 2). For EVOH1, absorbances of all representative peaks were higher for the pristine substrates versus all other substrates (90 d abiotic, and the 104 and 180 d biotic); similarly, the absorbances for all abiotic substrate peaks were greater than those of the 180 day biotic substrates (Fig. 1D; Table S5). The absorbances of four peaks (wavenumbers 1,330, 1,435, 2,855, 2,928, and 3,346 cm^−1^) were lower (*P* = 0.01) for the biotic 104 d substrate versus the abiotic (90 d) substrate (Fig. 1D; Table S5); however, there were no significant differences in absorbance across the wavenumbers (peaks) for the two biotic (104 and 180 d) samples (Fig. 1D; Table S5). The downward trend in mean absorbance values for all wavenumbers between pristine and abiotic (90 d) samples was similar to the trend between pristine and the 104 d biotic samples (not shown). This suggests that the changes in absorbances for all characteristic wavenumbers were likely due to abiotic hydrolysis and the plasticization of EVOH1 in water, with no biotic degradation evidenced.

**TABLE 2:** Representative ATR-FTIR peak assignments for EVOH (1, 2, and 3), PET, and CA

Polymer	Wavenumber (cm^−1^)	Assignment	Reference(s)
EVOH	833	Skeleton vibration	(52, 53)
	1,084	C-O stretching	(52, 53)
	1,330	O-H deformation	(52, 53)
	1,435	O-H deformation	(52, 53)
	2,855	C-H symmetric stretching	(52, 53)
	2,928	C-H asymmetric stretching	(52, 53)
	3,346	Hydrogen-bonded OH-stretching	(52, 53)
PET	730	Stretching vibration of the C = O-O-ester group	(54)
	1,100	Aromatic ester O-C-C stretch	(54)
	1,245	Aromatic ester C-C-O stretch	(54)
	1,510	Vibration aromatic skeleton with stretching	(54)
	1,715	Stretching of C = O carboxylic acid group	(54)
CA	1,050	C-O-C of the cellulose backbone	(55)
	1,240	C-O stretching of the acetyl group	(55)
	1,370	C-H bending vibration of CH_3_ in the acetyl group	(55)
	1,750	C = O stretching of the acetyl group	(55)

The trends in FTIR peak heights for EVOH2 differed somewhat from those for EVOH1. The skeleton peak (C-C; 833 cm^−1^) was significantly lower in the biotic conditions (T3 or 104 d, and T4 or 180 d) than in the 90 d abiotic and pristine control samples (*P* = 0.01; Fig. 1E; Table S5), and the 180 d biotic peak was significantly lower (*P* = 0.02) than the 104 d biotic peak (Fig. 1E; Table S5), suggesting that changes in the skeletal peak might be biotically driven. This was further evidenced as the downward trend in absorbance for this wavenumber across the pristine and two biotic samples was significantly linear over time, while the abiotic sample absorbance did not differ significantly from the pristine sample (Table S5; Fig. 1E). The ether (C-O; 1,084 cm^−1^) peak patterns were similar to those for the 833 cm^−1^ wavenumber with the exception that the abiotic sample significantly differed from the pristine sample (Table S5; Fig. 1E). The mean absorbances for the abiotic and two biotic samples changed similarly over time with respect to the pristine sample (Fig. 1E; Table S5). Therefore, changes in concentration of the C-O bond likely owed to abiotic processes. The alcohol O-H peaks (1,330 and 1,435 cm^−1^) were lower for the abiotic and biotic (104 d and 180 d) samples in comparison to the pristine control (*P* = 0.01, Fig. 1E; Table S5). The mean absorbance for the 1,435 cm^−1^ (but not the 1,330 cm^−1^) wavenumber was significantly lower in the 180 d biotic versus the 90 d abiotic (*P* = 0.01) and the 104 day biotic (*P* = 0.04; Table S5; Fig. 1E) samples. Yet, as there was no significant difference in mean absorbance between the 90 d abiotic and 104 day biotic (Fig. 1E; Table S5), the decrease in the alcohol peak is likely due to abiotic factors. This is supported by the absorbance trend over time: the abiotic and both biotic samples fell on a similar downward line of regression with respect to the pristine sample (not shown). For the alkane (2,855 and 2,928 cm^−1^) and hydrogen-bonded alcohol (O-H) group (3,346 cm^−1^) peaks, all the conditions (i.e., 90 d abiotic, T3 biotic, T4 biotic) differed from the pristine control (*P* = 0.01; Table S5; Fig. 1E). For the 2,855 cm^−1^ wavenumber, the abiotic absorbance was significantly greater than the 104 d biotic (*P* = 0.02, Table S5; Fig. 1E); otherwise, there were no significant differences for these three wavenumbers when comparing abiotic samples to biotic samples (104 d and 180 d) or the biotic samples to each other (Table S5; Fig. 1E), suggesting that the change in absorbances for those groups is likely due to abiotic hydrolysis and the plasticization of EVOH2 in water, with no evidence of biotic degradation.

Similarly to EVOH2, the skeletal (C-C; 833 cm^−1^) peak for EVOH3 was not significantly different when comparing the pristine sample to the abiotic (90 d) sample; however, the absorbances of the 833 cm^−1^ wavenumber for the biotic (104 d and 180 d) samples were significantly lower (*P* = 0.01) than the abiotic and pristine samples (Fig. 1F; Table S5). Furthermore, the downward trend in absorbances across the pristine and two biotic samples was significantly linear (not shown). Thus, similarly to EVOH2, this wavenumber evidenced biotic mechanisms in polymer changes over time. For all other wavenumbers (1,084, 1,330, 1,435, 2,855, 2,928, and 3,346 cm^−1^; Table 2), the pristine polymer absorbances significantly (*P* = 0.01) exceeded those of the abiotic and two biotic samples (Fig. 1F; Table S5). For one wavenumber, 1,084 cm^−1^, the abiotic sample absorbance exceeded (*P* = 0.02) that of the biotic 104 d sample (Table S5; Fig. 1F); however, the absorbances for all four samples fell along one significantly linear downward trendline, indicating that abiotic processes were likely explaining the decreases (not shown). For the other wavenumbers (1,330, 1,435, 2,855, 2,928, and 3,346 cm^−1^; Table 2), the similar absorbance values for the abiotic versus 104 d biotic samples (Table S5; Fig. 1F), considering the downward trend in absorbances from the pristine through the biotic 190 d samples (Fig. 1F), supported abiotic hydrolysis and the plasticization of EVOH3 in water as the likely mechanism of polymer change (29).

**Fig 2:**
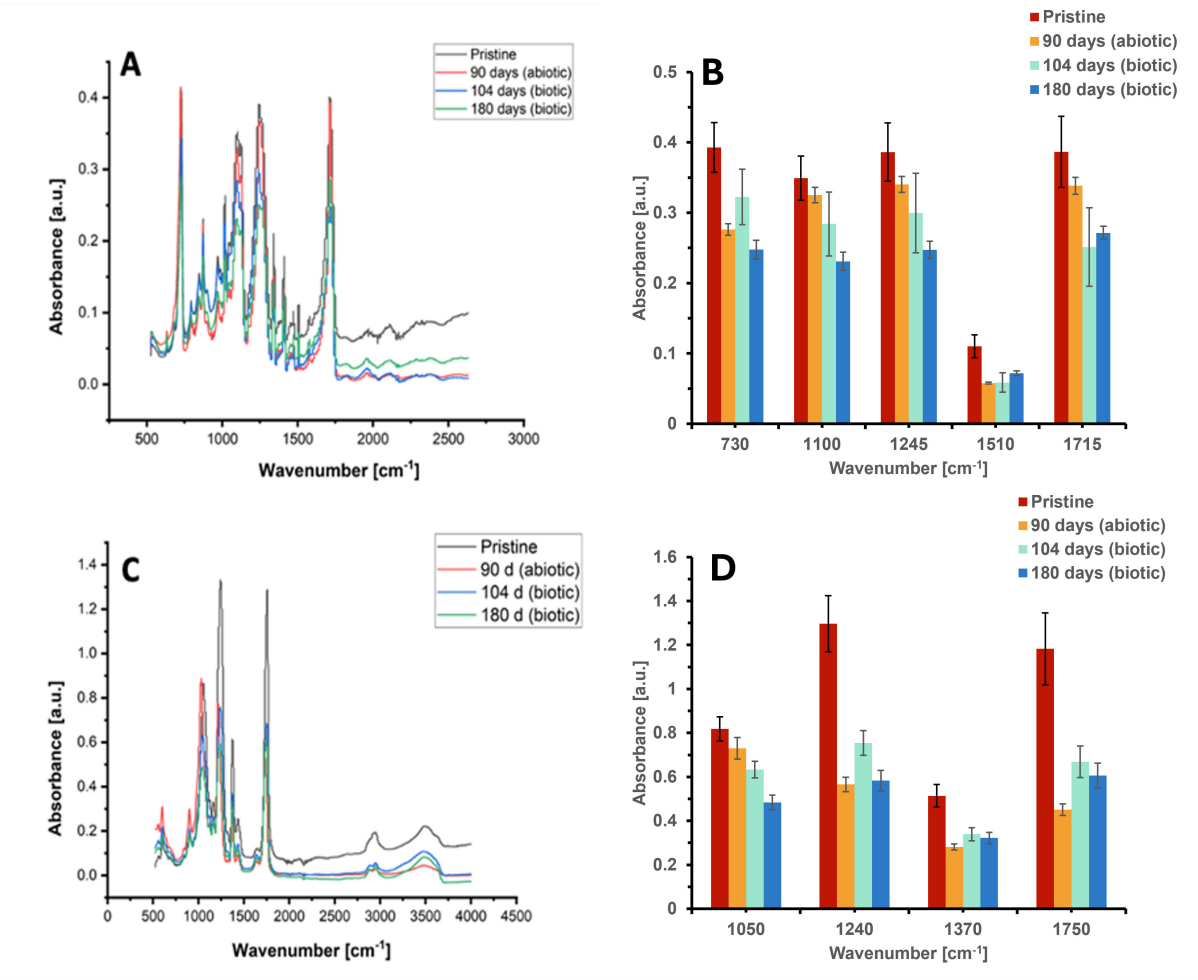
ATR-FTIR spectra (**A, C**) and average absorbances of the representative peaks (**B, D**) of the polymer substrate surfaces across all the relevant conditions in this study (A and B, PET; C and D, CA). Error bars are relative standard deviation.

The representative peaks for PET were 730 cm^−1^ (ester group), 1,100 and 1,245 cm^−1^ (terephthalate groups), 1,510 cm^−1^ (aromatic skeleton group; C = C), and 1,715 cm^−1^ (carboxylic acid group) (54) (Table 2). For all wavenumbers, the mean absorbances significantly differed (*P* < 0.05) between the pristine and 180 d biotic samples (Fig. 2B; Table S5). For the ester group (730 cm^−1^), there were no other significant differences in mean absorbances across the samples. For all wavenumbers except for the ester group (730 cm^−1^), the mean absorbance significantly differed between the abiotic sample and the 180 d biotic sample (Table S5; Fig. 2B, *P* < 0.05). One other significant difference (*P* = 0.01) was for the 1,510 cm^−1^ wavenumber absorbances when comparing the abiotic and pristine samples (Fig. 2B; Table S5). Therefore, based on ATR-FTIR results, there is limited abiotic and biotic degradation of the PET films. Despite there being no significant difference in comparison to the T3 104 d biotic condition, a decrease in concentration of the ester groups (730 and 1,100 cm^−1^) in the T4 180 d biotic condition could indicate potential esterase production by the PET plastisphere contributing to polymer degradation, while the concentration of the alkene groups remained unchanged (Fig. 2B).

The representative peaks for CA were specifically 1,050 cm^−1^ (cellulose backbone) and 1,240, 1,370, and 1,750 cm^−1^ (acetyl groups) (55) (Table 2). The mean absorbance of the cellulose backbone was decreased significantly (*P* < 0.05) when comparing the T4 (180 d) biotic samples to all other samples (Table S5; Fig. 2D). Furthermore, the T3 (104 d) sample absorbance for this wavenumber was significantly lower than for the pristine sample (Fig. 2D; Table S5). That the abiotic and pristine sample absorbances did not differ for this wavenumber, and that the downward trend between the pristine and biotic samples over time was more significantly linear than across all samples over time reinforced that there was biotic breakdown of cellulose, likely through cellulase production (Fig. 2D). However, the acetyl peaks (1,240, 1,370, and 1,750 cm^−1^) showed a different trend: all were significantly decreased (*P* < 0.05) when comparing the pristine control samples to the abiotic and 180 d biotic samples; additionally, the 1,240 cm^−1^ peak was significantly lower in the 104 d biotic sample compared to the pristine control (*P* = 0.01; Table S5; Fig. 2D). However, the 90 d abiotic, T3 biotic, and T4 biotic samples did not differ in their mean absorbances, indicating altogether that mainly abiotic factors resulted in the decreases (Table S5; Fig. 2D). The decrease in mean absorbances for the acetyl groups between the pristine and the biotic samples suggested that a deacetylation step, which is the initial degradation (and rate-determining) step, is occurring. Depending on the degree of substitution of the CA film substrate, deacetylation can happen by microbially produced acetylesterase attack or chemical hydrolysis (56, 57).

### No significant changes in thermal behavior of all substrate films

The thermal behavior of all substrate films exposed to the 90 d abiotic and T4 biotic conditions was characterized and compared to the pristine polymers to understand whether degradation is occurring to the bulk polymer (i.e., decomposition) and not only constrained to the surface. Decreased *T*_g_, *T*_m_, and *T*_c_ indicate degradation due to decreased molecular weight, breakdown in crystallinity, and increased mobility of shorter chains (58). Across all polymers and conditions, there were no significant differences (*P* > 0.05) in melting and crystallization temperatures, as well as degree of crystallinity, indicating that the bulk of the films remained intact (Table 1). Initial exploratory analyses focused on *T*_g_ at the harvest time intervals, but no differences were noted, and therefore the scan window for the T4 biotic and 90 d abiotic substrates focused on the crystallization and melting peaks for EVOH1, EVOH2, EVOH3, and PET (Table 1). DSC thermograms of the CA showed no crystallization and melting peaks; therefore, the degree of crystallinity and comparison of the crystallization and melting temperatures could not be used to infer crystallinity changes in the CA films.

### Bacterial communities varied by sample type and diverged over time

There was no extractable DNA on the sterilized plastic and glass coupons prior to being buried in the microcosm sediments, indicating that no bacteria were present initially and thus colonization was from the environment (data not shown). For the biotic experiment, the amount of extractable DNA, an indicator of biomass on the polymer and glass substrates (Table S6), indicated that very few microbes colonized the substrate surfaces.

**Fig 3:**
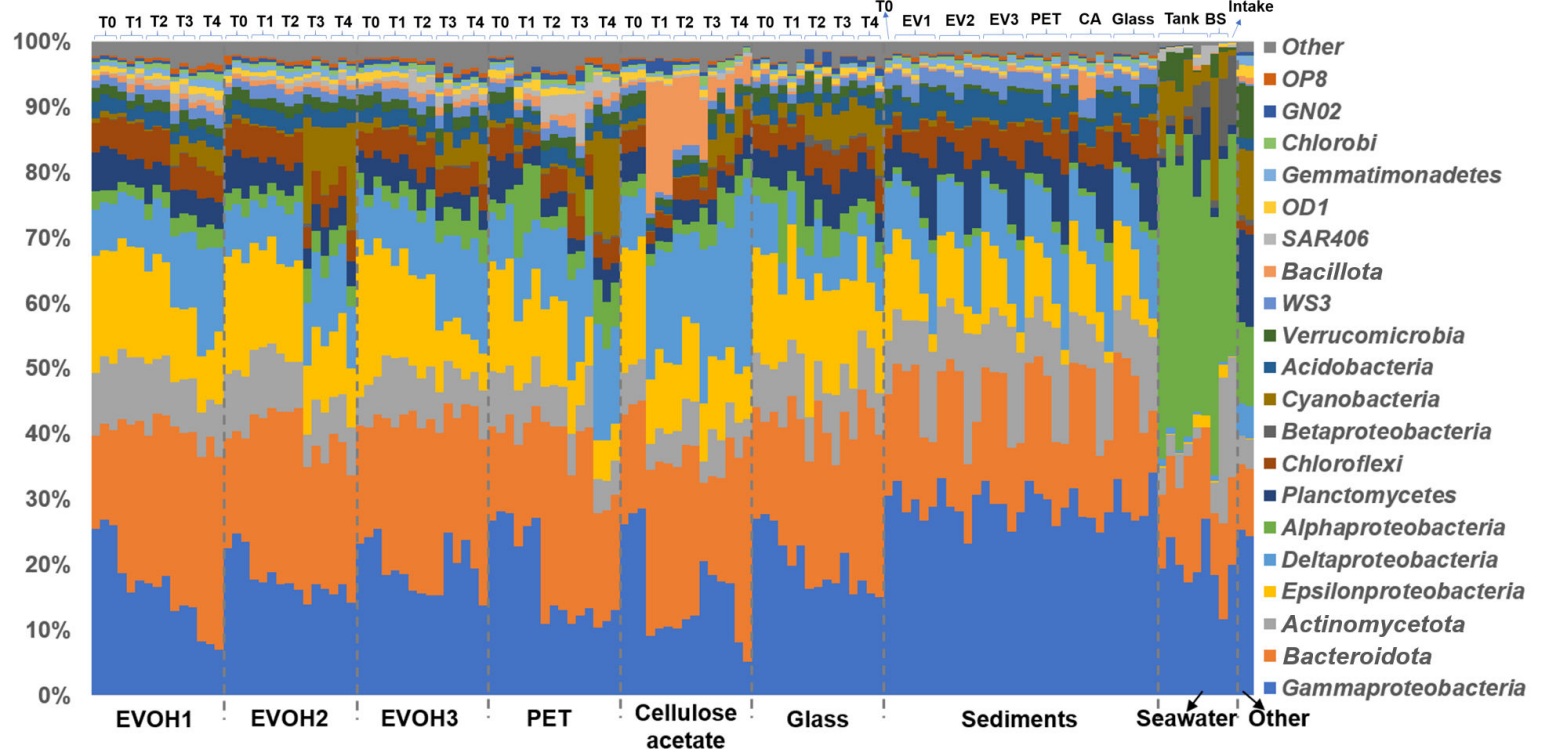
The relative abundance of the 20 most abundant bacterial phyla and superclasses in all samples of this study (EVOH1, *n* = 15; EVOH2, *n* = 15; EVOH3, *n* = 15; PET, *n* = 15; CA, *n* = 15; glass, *n* = 15; sediments, *n* = 31; seawater, *n* = 9; tank surface samples, *n* = 2). The percentage on the y-axis denotes the percent of the total sequence abundances. From left to right (EVOH1 through 3, PET, cellulose acetate or CA, and glass), each clustered bar on the x-axis denotes a replicate and corresponds to the order of biotic experiment sampling times from T0 to T4 for the harvested substrates. For the sediment samples, the first clustered column was the homogenized sediments used to fill the microcosms before incubation (T0), followed by the sediments collected from microcosms for each kind of substrate in the order of EVOH1 (EV1; *n* = 5), EVOH2 (EV2; *n* = 5), EVOH3 (EV3; *n* = 5), PET (*n* = 5), cellulose acetate (CA; *n* = 5), and glass (*n* = 5). From left to right, each clustered bar on the x-axis denotes a replicate and corresponds to the order of sampling times from T0 to T4 for the substrate-containing sediments. For the seawater, one sample was taken from the incubation tank at each sampling timepoint (Tank, T0 to T4, *n* = 5), 3 samples from Brian Seep (BS; T0, 2 days after T4, and 9/24/21), as well as one sample from the seawater system intake location 2 days after T4. The other (Other) two samples were tank surface samples that were scraped from the bottom and side of the incubation tank at T4. Note that “Other” in the taxon legend refers to sequences unassigned to phyla or superclasses.

The most abundant groups at the phylum and superclass taxonomic levels found on all of the plastic and glass coupons from T0 to T4 of the biotic experiment were Bacteroidota, Gammaproteobacteria, Epsilonproteobacteria, Deltaproteobacteria, Actinomycetota (formerly Actinobacteria), Chloroflexi, Planctomycetes, and Alphaproteobacteria, and the relative abundances were individually 12.7%, 10.1%, 7.3%, 6.2%, 4.1%, 2.3%, 2.3%, and 2.0% on average, respectively (Fig. 3). The communities clearly varied on each kind of substrate and became gradually divergent among the substrates. The relative abundance of Gammaproteobacteria decreased on all substrates from T0 to T4, while the relative abundance increased for Bacteroidota and Cyanobacteria (Fig. 3). Compared to other substrates, the relative abundance of Bacillota increased over time on the CA substrates. At T0, the average relative abundance of Bacillota was 0.5%, which later increased to 5.4% at T4 (Fig. S6). None of the other substrates had a Bacillota relative abundance exceeding 0.7% (Fig. S6).

The most abundant taxa found in all microcosm-sampled sediments across all time points were Gammaproteobacteria, Bacteroidota, Actinomycetota, Deltaproteobacteria (Desulfobacterota), Epsilonproteobacteria, Planctomycetes, Chloroflexi, and Acidobacteria (Fig. 3). These phyla are typically found in hydrocarbon-rich sediments and are known to have genes involved in anaerobic hydrocarbon degradation (23). Although the bacterial communities in sediments for each substrate also evolved clearly from T0 to T4, the communities in different substrate microcosms were generally similar at each sampling point. However, similarly to those on substrates, Bacillota also became remarkably abundant in sediments of microcosms for cellulose acetate, with the relative abundance of 1.4% during T1–T4, in contrast to 0.16% in sediments for other substrates during T0 to T4 and cellulose acetate at T0.

Compared to those on substrate surfaces and in sediments, the dominant bacterial group in all seawater samples was Alphaproteobacteria with a relative abundance of 28.4% over time (Mann–Whitney Test, all *P* < 0.001, *n* = 129). Other dominant bacterial groups in the seawater collected from the microcosm study tank were primarily Gammaproteobacteria, Bacteroidota, Cyanobacteria, Planctomycetes, Betaproteobacteria, Actinomycetota, and Verrucomicrobia with an average relative abundance over time of 13.3%, 10.1%, 4.4%, 2.1%, 1.8%, 2.2%, and 2%, respectively (Fig. S7). In comparison to all substrate and sediment samples, Verrucomicrobia were more abundant in seawater samples, which coincides with past studies where their abundance is highest in coastal ocean water and lowest in sediments from hydrothermal vents (59). In contrast, the relative abundances of certain bacterial hydrocarbon-degrading groups, such as Epsilonproteobacteria, Deltaproteobacteria, and Chloroflexi, were lower in the seawater samples (less than 0.5%) than those on substrates and in sediments. The two scraping samples from the incubation tank at T4 showed clearly different bacterial communities from those in seawater and sediments (Fig. 3).

**Fig 4:**
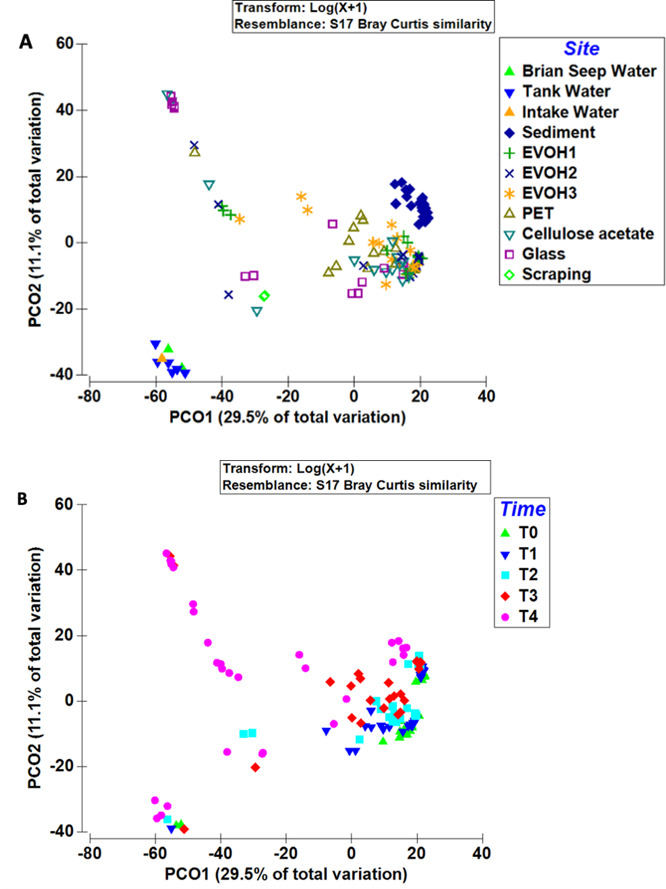
PCoA of the bacterial communities based on the Bray–Curtis distance of the transformed (log(x + 1)) relative abundance of bacterial species in each sample of this study (**A**) including EVOH1, EVOH2, EVOH3, PET, CA, and borosilicate glass substrates, homogenized sediment prior to microcosm setup (T0) and the substrate-containing sediments from microcosms (T1–T4), seawater from the incubation tank (Tank Water), Brian Seep Water, and seawater system Intake Water, and tank surface (Scraping). Bacterial communities in all seawater and sediments were separated (ANOSIM test, Global *R* = 1, *P* = 0.001; PERMDISP test, *P* = 0.12). Bacterial communities on five different kinds of plastic and glass substrates showed more similarities to sediment communities (ANOSIM test, Global *R* = 0.013, *P* = 0.39) than seawater communities (ANOSIM test, Global *R* = 0.488, *P* = 0.001; PERMDISP test, *P* = 0.10); and (**B**) based on the different sampling timepoints from T0 to T4 of these samples. The seawater samples taken from Brian Seep and the seawater system intake 2 days after T4 were also marked as T4 for simplicity. All sampling time (T0–T4) point substrates (EVOH1, EVOH2, EVOH3, CA for cellulose acetate, PET, and glass), and sampling locations (BS for Brian Seep) are represented in part A of the figure.

PCoA (Fig. 4) further confirmed the above observations. Bacterial communities in all seawater and sediments were separated (ANOSIM test, Global *R* = 1, *P* = 0.001; PERMDISP test, *P* = 0.12), and further different from those on substrates. Bacterial communities on five different kinds of plastic and glass substrates showed more similarities to sediment communities (ANOSIM test, Global *R* = 0.013, *P* = 0.39) than seawater communities (ANOSIM test, Global *R* = 0.488, *P* = 0.001; PERMDISP test, *P* = 0.10). Furthermore, communities at T0 on all substrates were already significantly different from those in sediments and seawater at T0 (ANOSIM test, Global *R* = 0.976, *P* = 0.001, all pairwise tests *R* > 0.967, *P* < 0.03; PERMDISP test, *P* = 0.18), indicating that selective bacterial colonization (i.e., enrichment) on substrate surfaces occurred rapidly. Bacterial communities on substrate surfaces and in sediments and seawater all showed clear changes from T0 to T4, and became more divergent at T3 and T4 on plastic and glass substrates.

The average Chao1 richness values of bacterial communities on all substrates (2.6E + 03) were significantly higher than those of bacterial communities in sediments (1.4E + 03) (Mann–Whitney Test, *P* < 0.001, *n* = 121), and higher than that of seawater (2.0E + 03) (*P* > 0.05, *n* = 99) (Table S6; Fig. S8). The bacterial community richness values for all plastic and glass substrates increased from T0 to T4 (Fig. S8), further confirming that bacterial communities on the substrates became increasingly complex over time.

SourceTracker results indicated that bacteria on the plastic and glass substrates primarily originated from the sediments, with estimated average proportions of 65.6% (Table S6). In contrast, the estimated contributions of bacterial communities from seawater were 0.9%. There were no observed significant differences in the contributions from either source (i.e., sediments or seawater) between the plastic and glass substrates.

### Dominant core bacterial genera on all substrates

**Fig 5:**
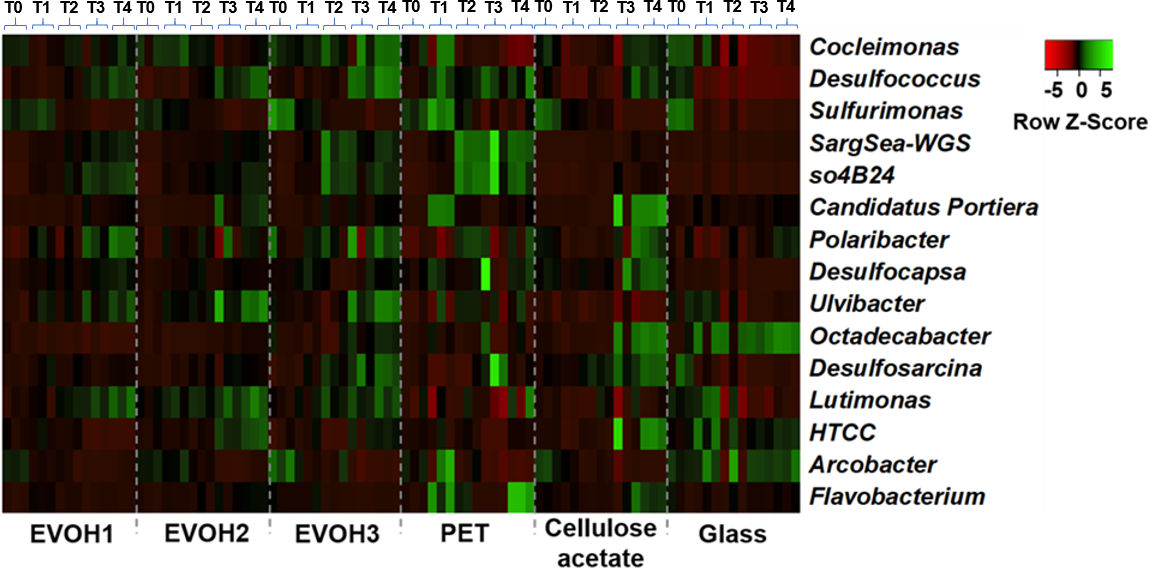
A heatmap of the relative abundances of bacterial core genera prevalent on all substrates in this study, that is, EVOH1 (*n* = 15), EVOH2 (*n* = 15), EVOH3 (*n* = 15), PET (*n* = 15), cellulose acetate (*n* = 15), and glass (*n* = 15). The top 15 bacterial genera with the highest average relative abundance were selected and are shown in order from top to bottom. The relative abundance of the bacterial genera on plastic polymers and glass is presented in order of sampling time from T0 to T4, as shown at the top of the figure. The coloring as per the legend: green for the highest and red for the lowest. Bacterial genera are displayed according to their relative abundances (normalized by Z-score across all data sets).

Among the core bacterial genera discovered on the substrates, the 15 most dominant core bacterial genera on all plastic and glass substrates included *Cocleimonas*, *Desulfococcus*, *Sulfurimonas*, *SargSea-WGS*, *so4B24*, *Candidatus* Portiera, *Polaribacter*, *Desulfocapsa*, *Ulvibacter*, *Octadecabacter*, *Desulfosarcina*, *Lutimonas*, *HTCC*, *Arcobacter*, and *Flavobacterium* (Fig. 5; Table S6). While some of the genera were also abundant in sediments or seawater, the relative abundances of most of these core bacterial genera—specifically *Desulfococcus*, *SargSea-WGS*, *so4B24*, *Candidatus* Portiera, *Polaribacter*, *Desulfocapsa*, *Ulvibacter*, *Octadecabacter,* and *Desulfosarcina*—increased on the substrates during incubation from T0 to T4, suggesting that these bacterial genera were important members of adherent bacteria growing on the substrates. Furthermore, most genera, such as *Desulfococcus*, *Sulfurimonas*, *SargSea-WGS*, *so4B24*, *Polaribacter*, *Desulfocapsa,* and *Desulfosarcina* were significantly relatively more abundant on all plastic substrates as compared to the glass controls.

### Bacterial genera were enriched on polymers, in tank water, and in sediments

Bacterial genera enriched on polymers were important adherent bacterial community components. Most bacterial genera enriched on the surface of plastic or glass substrates, such as *so4B24, Sphingomonas, Mycobacterium, Agromyces, Brevundimonas, Corynebacterium, Desulfococcus, SargSea-WGS, Sphingopyxis,* and *Delftia* (Table S7), were shared across one or more substrates. Furthermore, many of these enriched groups were also dominant core bacterial genera, such as *Desulfococcus, SargSea-WGS, so4B24, Candidatus* Portiera*, Polaribacter, Ulvibacter, Octadecabacter, Lutimonas,* and *Flavobacterium*, indicating that all of the above genera were possibly important components of the adherent bacterial communities.

Bacterial genera enriched in the tank water were common residents of marine waters, such as *Octadecabacter, Polaribacter, ZA3312c, Winogradskyella,* and *Loktanella* (Table S7). Meanwhile, several bacterial genera, such as *Desulfococcus, Desulfosarcina, Desulfocapsa, Planctomyces, C1_B004, MSBL3,* and *Desulfomonile*, were enriched for in the sediments containing the various substrates, confirming that the bacterial communities in the sediments were generally similar at each sampling time point.

### Few bacterial genera were significantly associated with polymers, except for CA

Bacterial genera significantly more abundant in seawater and sediments were identified using the LEfSe and the DESeq2 methods (Fig. S9). Compared to sediments, bacterial genera significantly associated with seawater were *Candidatus, Portiera*, *Octadecabacter*, *Synechococcus*, *Flavobacterium,* and *Loktanella*, all of which are common residents in marine waters (Fig. S9). Bacterial genera that were abundant in sediments in comparison to seawater were mainly anaerobes, including *Cocleimonas*, *Desulfococcus*, *Spirochaeta*, *Desulfocapsa,* and *Thiocapsa* (Fig. S9), most of which are involved in sulfur cycling, such as sulfur-oxidizing *Cocleimonas* sp. bacteria, sulfide-oxidizing *Thiocapsa* sp. bacteria, and sulfate-reducing *Desulfococcus* spp. and *Desulfocapsa* spp. bacteria. This is likely because the microcosm sediments were sourced from suboxic sediments in a hydrocarbon seep.

In comparison to seawater and sediments, some bacterial genera, namely *Fusibacter*, *Mycobacterium, SargSea-WGS*, *so4B24,* and *Arctic95A-2,* were more abundant on EVOH1 through 3 and PET than on CA during T1 to T4 (Mann–Whitney Test, all *P* < 0.05, *n* = 72). *Sulfurimonas* and *Psychromonas* were also more abundant on the plastic substrates than in seawater and sediments (Mann–Whitney Test, both *P* < 0.001, *n* = 132), but gradually decreased during incubation from T0 to T4, suggesting that they might be among the initial bacterial colonizers on the plastics.

**Fig 6:**
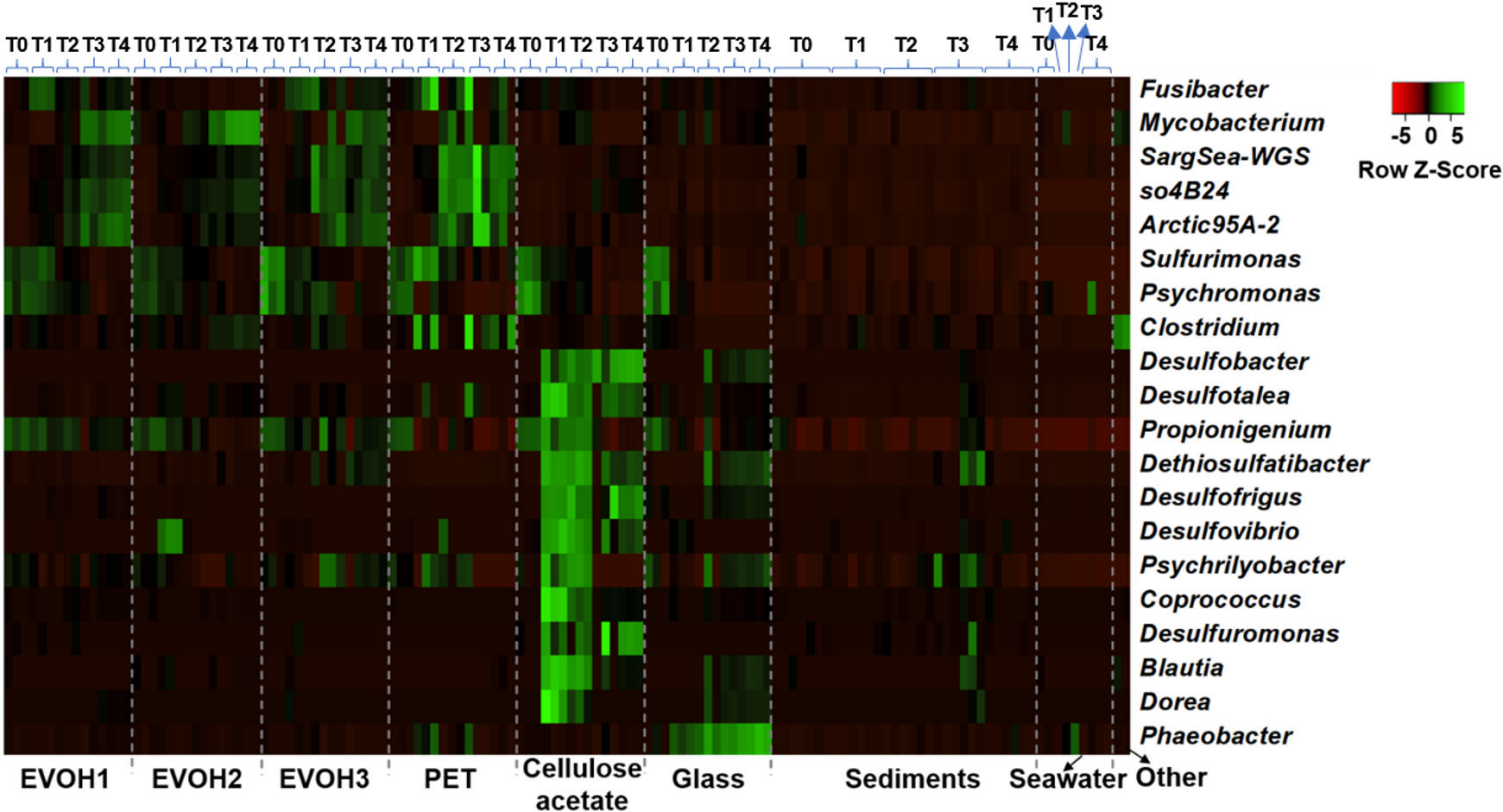
Heatmap of relative abundances out of all sequences of bacterial genera significantly associated with all substrates in comparison to the sediments and seawater. The samples included EVOH1 (*n* = 15), EVOH2 (*n* = 15), EVOH3 (*n* = 15), PET (*n* = 15), cellulose acetate (*n* = 15) and glass (*n* = 15) substrates, sediments (*n* = 31), seawater (*n* = 9), and tank surface samples (*n* = 2). From left to right, the samples of all substrates are in the order of sampling time from T0 to T4, as shown at the top of the figure. For the sediment samples, the first column was the homogenized sediments used to fill the microcosms before incubation (T0), followed by the sediments collected from substrate-containing microcosms from T0 (*n* = 5) to T4 (*n* = 5) represented by each clustered bar on the x-axis. Within each sampling time, the samples are in the order of substrates EVOH1, EVOH2, EVOH3, PET, cellulose acetate, and glass from left to right. Other refers to the tank scraping (surface) samples. The coloring as per the legend: green for the highest, and red for the lowest, relative abundance. Bacterial genera are displayed according to their relative abundances (normalized by Z-score across all data sets).

Notably, however, when comparing bacterial communities across the different substrates, no bacterial genera were identified that were significantly associated with any of the EVOH substrates. Only one genus, *Clostridium,* was significantly associated with PET (Fig. 6). In contrast, a number of genera were significantly associated with CA (Fig. 6), mainly including *Desulfobacter, Desulfotalea, Propionigenium, Dethiosulfatibacter, Desulfofrigus, Desulfovibrio, Psychrilyobacter, Coprococcus, Desulfuromonas, Blautia,* and *Dorea*, suggesting that the CA enriches for more bacterial groups, including possible biodegraders. Lastly, the bacterial genus *Phaeobacter*, which is a noted biofilm-forming microbe on marine surfaces (60), was more abundant on the borosilicate glass control than on plastic substrates.

When comparing bacterial communities in sediments collected from microcosms incubated with different substrates from T1 to T4, no bacterial genera were significantly associated with sediments that contained any plastic or glass substrates, except for CA. Bacterial genera that were significantly more abundant in sediments with CA were similar to clades on cellulose acetate substrates, mainly including *Desulfobacter, Desulfotalea, Dethiosulfatibacter, Coprococcus,* and *Blautia*.

### Potential plastic biodegrader species

By comparing the bacterial species found in this study with those listed in a plastic-biodegrading bacterial database (6), more potential biodegrader species were observed on polymers than in sediments and marine water. Overall, the number of potential biodegrader species was 12 on polymers, 6 in sediments, and 3 in marine water (Table S6). The number of plastic biodegrader species on polymers generally increased during incubation, with 4 at T0, 5 at T1, 8 at T2, 6 at T3, and 6 at T4 (Table S6). Furthermore, the number of biodegrader species was 4 on EVOH1, 8 on EVOH2, 9 on EVOH3, 5 on PET, 6 on CA, and 5 on glass (Table S6).

## DISCUSSION

In this study, natural marine sediments from a methane seep with their intact bacterial communities were studied for biotic and abiotic fates of various plastic substrates. The combined physicochemical analyses of the plastic substrates including surface heterogeneity by SEM and image analysis, surface hydrophobicity by goniometry, functional groups by ATR-FTIR, and the glass transition temperature (*T*_g_), melting temperature (*T*_m_), the crystallization temperature (*T*_c_), and the degree of crystallinity by DSC indicated that almost no degradation occurred for three types of EVOH or PET, while CA was degraded to a certain extent. The inference of surface biodegradation was mainly from analysis of ATR-FTIR data, producing scant evidence for EVOH biodegradation and more definitive evidence for CA biodegradation.

Hydrophobicity of the plastic surfaces was determined using contact-angle goniometry, and surface roughness is expected to influence the contact angle (61). Specifically, increased surface roughness will decrease the contact angle on hydrophilic surfaces (<90°) but increase the contact angle on hydrophobic surfaces (>90°) (61, 62). Therefore, the rougher the surface, the higher the surface energy due to a higher effective area, which, in turn, contributes to the hydrophilicity of the polymer (61, 63). Additionally, increased hydrophilicity is conducive to microbial attachment and colonization, which could, in turn, contribute to a faster degradation rate (63). Despite the apparent contact angle of CA and PET being less than 90°, both polymers did not follow the assumption that increased surface roughness prompts a decrease in their contact angle. This could be because the droplets on the polymer surface are not in a Wenzel state; that is, there was no air between the polymer and the droplet. Instead, they could be in a Cassie-Baxter state, whereby air is trapped in the grooves of surface features, in turn forming an air–solid composite overall-hydrophobic surface, resulting in the contact angle increasing (64, 65). Therefore, to evaluate both surface roughness and hydrophilicity, confirmation of this difference is crucial either with SEM micrographs, as done in this study, or with atomic force microscopy.

The dissimilarity of bacterial community composition that was observed between substrate surfaces and in sediments at T0 during this study suggested that bacterial colonization of plastic debris in sediments can happen within hours, as reported previously (17). The overall increase in the relative abundance of Bacteroidota and decrease in Gammaproteobacteria was observed on all plastic and glass substrates (Fig. 3). Gammaproteobacteria have been characteristic of primary biofilm colonizers in previous studies, while Bacteroidota are known as secondary colonizers in marine environments, with a similar phenomenon of community shift from primary to secondary colonizers reported previously (66, 67). The overall bacterial community successions were clearly observed on all plastic and glass substrates (Fig. 4; Table S6), particularly between T3 and T4. Previous studies indicated that microbial communities on plastic surfaces were substrate-dependent (68, 69), while some research also observed similar communities on different kinds of plastics (18, 70). In this study, communities on substrates became more divergent during incubation, suggesting that bacterial communities were substrate-dependent in the marine sediments.

During incubation, bacterial communities on plastic and glass substrates became increasingly different from those in sediments and seawater (Fig. 4), as reported previously (17, 18). Most of the bacterial species on substrates of this study originated from sediments rather than seawater, as supported by SourceTracker results. An additional control study that incubated substrates within only seawater could have indicated if bacterial taxa enrichment from seawater onto polymers differed from sediments. Furthermore, sediment pore water analysis of bacterial communities could be of interest for distinguishing particle-associated versus planktonic inocula governing substrate colonizers. However, this study focused on sediments, inclusive of particle- and pore water-associated sources of inocula onto the substrates. Previous studies indicated similar or lower richness levels of microbial communities on plastics than in sediments, while similar richness levels appeared between plastics and seawater (18, 19). In this study, the richness of communities on plastics was higher than that of sediments, but similar to that of seawater, suggesting that enrichment on substrates was owed to a favorable habitat for many taxa. Consistent with a previous study (71), the richness of communities on all plastic and glass substrates increased gradually, confirming that bacterial communities on substrates became more complex during incubation.

The overall bacterial communities on substrates here were generally different from those reported at different geographical locations (17, 19, 72), providing additional evidence that geographic location of the inoculum is one of the major factors impacting microbial community composition on plastic surfaces (70, 73). Brian Seep in the Santa Barbara Channel, California, is known for organic-rich sediment accumulation and hydrocarbon (primarily methane) seafloor venting (Table S2) (27, 28). This might result in the predominance of Epsilonproteobacteria and Deltaproteobacteria, including sulfur cycling bacteria such as sulfur-oxidizing, sulfide-oxidizing, and sulfate-reducing bacteria in such petroleum gas-rich sediment environments.

Sulfate-reducing bacteria such as *Desulfococcus*, *Desulfocapsa*, and *Desulfosarcina* were further prevalent and significantly more abundant on all plastic substrates in this study as compared to glass controls, suggesting that these potential petroleum gas biodegraders might also degrade plastics slowly or utilize chemicals leaching from plastics. Meanwhile, other core bacterial members prevalent on all plastic substrates (Fig. 5) may have been important bacterial community components, utilizers of the leaching chemicals, or even biodegraders of plastics. For example, *Polaribacter* sp. and *Flavobacterium* sp. bacteria belonging to Bacteroidota are considered to be late colonizers on plastics (19, 74). *Fusibacter* was observed to be enriched in sediments mixed with polyethylene (PE), polyvinyl chloride (PVC), polyurethane (PU), and polylactic acid (PLA) microplastics (75). Additionally, *Fusibacter* has been identified as a dehalogenating genus, involved in the degradation of PVC via reductive dechlorination of tetrachloroethene to cis-dichloroethene (76). *Mycobacterium* is a known genus of aerobic polyaromatic hydrocarbon degraders, which may explain its abundant colonization of synthetic plastics (77). Additionally, *Mycobacterium* sp. play a role in the degradation of PET paired with other organisms within biofilm (such as *Rhizopus* sp.), which cleave the ester bond of PET into bis(2-hydroxyethyl) terephthalate (BHET) that is then further decomposed by *Pseudomonas* sp. into monomeric units of terephthalate and ethylene glycol and further assimilated by *Mycobacterium* sp. (78). *Mycobacterium* sp. in seawater have also been shown to aerobically degrade polystyrene (PS), by the oxidation of the vinyl side chain (79) or anaerobically by use of styrene as the sole carbon and energy source (80). *Ulvibacter* sp. bacteria were specifically associated with different types of plastics and played a significant, but undefined, role in biodegradation (70). *Sulfurimonas* sp. bacteria were found to be dominant colonizers on PE plastic debris after two decades in deep-sea sediments at a water depth of 4,150 m (81). Some core bacterial genera in this study, such as *Octadecabacter*, *Phaeobacter*, *Amaricoccus,* and *Dinoroseobacter* (Table S6), belong to the Rhodobacteraceae, which are usually core members of biofilm on plastics (66, 82). Additionally, several enriched bacterial genera, including *Sphingomonas*, *Sphingopyxis*, *Novosphingobium*, and *Sphingosinicella,* are commonly referred to collectively as sphingomonads, which can metabolize a wide variety of refractory carbon sources and thus survive in low-nutrient conditions. All of these results indicated that the bacterial groups prevalent or enriched on plastic substrates in this study were important adherent components, utilizers of leaching chemicals, and even potential biodegraders of plastics, many of which were sourced from the Brian Seep sediments.

When comparing bacterial communities on different substrates, some bacterial genera significantly associated with each plastic substrate could specifically be biodegraders. In this study, fewer bacterial genera were identified to be significantly associated with conventional plastics in comparison to cellulose acetate (CA). This is further supported by the physicochemical analyses of the plastic substrates in this study, which suggested very little biodegradation occurring to EVOHs and none to PET, but a certain extent of biodegradation occurring for CA as evidenced by ATR-FTIR results for the cellulosic backbone of CA (1,050 cm^−1^, Fig. 2D). Although no *Clostridium* sp. strains have been identified to degrade PET, *Clostridium* sp. strains degrading the natural polyesters poly(β-hydroxybutyrate) (PHB) and the synthetic polyester poly(ε-caprolactone) (PCL) were isolated from two anaerobic sludges (83), and the hydrolysis activity of the esterase from *Clostridium botulinum* on PET was identified and improved by concomitant engineering of two different domains (84). In contrast to conventional plastics, a number of bacterial genera were significantly abundant on surfaces of CA, mainly including sulfate-reducing bacteria such as *Desulfobacter*, *Desulfotalea*, *Dethiosulfatibacter*, *Desulfofrigus*, *Desulfovibrio,* and *Desulfuromonas*, the bacteria of the class Clostridia, such as *Dethiosulfatibacter*, *Coprococcus*, *Blautia,* and *Dorea*, as well as *Propionigenium* and *Psychrilyobacter* that belong to the phylum Fusobacteria. Sulfate-reducing bacteria were also abundant on CA buried in coastal sediments (72), and further dominant on biodegradable plastic polyhydroxyalkanoate (PHA) but not on PET in a coastal marine sediment in the northern Gulf of Mexico (85). The further analyses indicated that these sulfate-reducing bacteria contained genes integral to both biodegradable plastic degradation and sulfate reduction, and biodegradable plastics can promote a rapid and significant shift in benthic microbial diversity and gene pools, selecting for microbes that participate in biodegradable plastic degradation and sulfate reduction (85). The overall dominance of sulfate-reducing genera with CA (Fig. 6) demonstrates that the sulfate-reducing bacteria that originated from sediments may contribute to the biodegradation of CA. Potentially cellulolytic Clostridia (86), such as *Dethiosulfatibacter*, *Coprococcus*, *Blautia,* and *Dorea,* were usually observed in the gut microbiome and significantly enriched on CA surfaces in marine sediments (72). The significant association of these Clostridia with CA in this study confirmed that they might be able to degrade CA by the enzymatic attack of cellulose. Although CA is regarded as a biodegradable material, its degradation rate is highly influenced by the actual degradation environment (87). Biodegradation of plastics in anoxic or anaerobic sediments is suggested to be very slow (15), which was confirmed by the observation that only CA polymers among all the polymers in this study showed very limited changes of physiochemical characteristics, even if more bacterial genera were significantly associated with CA.

Beyond polymer type, plastic additive chemistry represents another critical factor shaping microbial colonization. Antimicrobial additives can select for resistant taxa, while other additives like surfactants can modify surface properties such as hydrophobicity (88), which, in turn, influence bacterial attachment. Certain additives may leach and serve as carbon sources for taxa with appropriate degradation enzymes (89, 90), while other additives at high concentrations can inhibit colonization (91) as elucidated through benchtop studies. Moreover, temporal shifts in additive leaching create dynamic chemical gradients that are likely to drive microbial community succession. *In situ* studies to date indicate that environmental factors exert stronger influences on plastic-associated microbial communities than polymer type alone (92–94). While this study did not characterize the leachable additives of these plastics, investigating their presence would provide deeper insight into the community assembly processes to inform the microbial community dynamics on environmentally relevant plastics.

Although sequencing genes encoding 16S rRNA effectively demonstrated that CA was the only material among the tested polymers to harbor specific bacterial taxa, a finding further corroborated by the physicochemical analyses, biodegradation cannot be conclusively determined from these data sets. Metagenomics, metatranscriptomics, or proteomics would be needed to fully evaluate the plastic-degrading abilities of the studied bacterial communities (95–97). Furthermore, the potential presence of non-bacterial microorganisms such as fungi or diatoms within the sediment may also be responsible or contribute to the potential degradation of plastics (18–21).

Consistent with a previous study by Vannini et al. (18), conventional plastics showed no significant impacts on the bacterial communities in marine sediments. However, CA exhibited a distinct pattern: several bacterial genera that were significantly more abundant on CA surfaces were also enriched in the adjacent sediments. This substrate-specific influence on both biofilm and sediment communities was unique to CA and not observed with petroleum-based plastics, revealing a likely fundamental difference in how biodegradable and conventional plastics affect surrounding microbial ecosystems.

## Data Availability

The sequencing data were deposited in NCBI SRA with the BioProject ID PRJNA1280069.

## SUPPLEMENTAL MATERIAL

Supplemental material aem.02490-25-s0001.pdf Supplemental methods, Tables S1 to S4, and Fig. S1 to S9.

Additional supplemental tables aem.02490-25-s0002.xlsx Tables S5 to S7.

ASM does not own the copyrights to Supplemental Material that may be linked to, or accessed through, an article. The authors have granted ASM a non-exclusive, world-wide license to publish the Supplemental Material files. Please contact the corresponding author directly for reuse.
