# Supplementary material for: Bacterial community colonization with minimal alteration of plastics in suboxic sediments from a marine methane seep
Source: Appl Environ Microbiol. 2026 Jun 4;92(7):e02490-25. doi: 10.1128/aem.02490-25 (PMC13390349; doi:10.1128/aem.02490-25)
Supplement: Supplemental material — Supplemental methods, Tables S1 to S4, and Fig. S1 to S9. [file aem.02490-25-s0001.pdf]

Supplemental Material

Bacterial Community Colonization with Minimal Alteration of Plastics in Suboxic Sediments from a Marine Methane Seep

Timnit Kefela,[a,e] Dong Li,[a] Laurie C. Van De Werfhorst,[a] Rebecca M. Reynolds,[a] Rachel Behrens,[c] Yuki Floyd,[a] , Jennifer L. Brown,[b] Jen Smith,[d] Michelle A. O'Malley,[b] Patricia A. Holden[a]#

[a]Bren School of Environmental Science & Management, University of California, Santa Barbara
[b]Department of Chemical Engineering, University of California, Santa Barbara
[c]Materials Research Laboratory, University of California, Santa Barbara
[d]California NanoSystems Institute, University of California, Santa Barbara
[e]Environmental Science and Resource Management Program, California State University, Channel Islands

*Corresponding author:
P. A. Holden
E-mail: holden@bren.ucsb.edu

Water and Sediment Sample Collection

Water and sediment samples for establishing *ex situ* microcosms were collected from an area of active bubble plumes at Brian Seep (34°N 24.109'; 119°W 49.917', water depth 10-11 m), a methane seep in the Santa Barbara Channel, on 12/16/2020 (the day of the microcosm establishment) by scientific divers. Water samples were collected using a 5 L Van Dorn bottle, and sediment using sterile 2 L or 4 L polypropylene bottles that were opened at the surface and filled with ocean water prior to descent. For water collection, the divers descended with both ends of the Van Dorn bottle open and flushed the interior of the bottle by moving it side to side before positioning over an active bubble plume. The bottle was held vertically over a bubble plume for at least 10 seconds before snapping the ends closed to capture the sample *in situ*. For sediment collection, the divers pushed the open bottles at a slight angle into the sediment (capturing ~0 to 15 cm depth) and then moved them horizontally to fill with sediment, repeating until the bottles were almost full. Additional sediment was scooped into the bottles using their caps until the bottles were completely full, and excess ocean water was displaced. Upon return to the boat, water samples were transferred to sterile 4 L polypropylene bottles, and physical parameters (dissolved oxygen, salinity, pH, and temperature) of water and sediment samples were measured for a subsample, using an HQ40d multiparameter meter (Hach, Loveland, OH).

Additional sediment samples were collected from Brian Seep on 9/24/2021 for C1-C4 hydrocarbon analysis to characterize the hydrocarbon chemistry of the sediment sampling environment. Triplicate 40 mL volatile organic analysis (VOA) glass vials were prepared at the surface (threads wrapped with Teflon tape, vials filled with seawater and then capped) and carried to the seafloor at the seep by scientific divers. Five active bubble plumes were selected, and at each plume the triplicate glass vials were uncapped and pushed into the sediment (capturing ~0 to 5 cm depth) and moved horizontally until they were ~75% full of sediment and 25% of surrounding seawater. The vials were then tightly closed at the seafloor; sampling at all bubble plumes was performed before ascent. Upon return to the boat, the vials were placed immediately on ice until same-day transport to Atmospheric Analysis & Consulting, Inc. (Ventura, CA), where the seawater was removed using syringes to create a headspace which was then analyzed for volatile hydrocarbons using EPA method RSK-175.

Substrate, Water and Sediment Characteristics

The pristine (unused) polymer and glass substrate characteristics were tabulated from manufacturer data (Table S1). The polymer substrates that were harvested from the microcosms at each time point for physicochemical characterization were first cleaned by rinsing and vortexing in Nanopure water (Methods, main manuscript), then analyzed to assess potential material changes indicative of degradation. Specifically, scanning electron microscopy (SEM) with micrograph image analysis was used to assess surface roughness differences related to pitting; differential scanning calorimetry (DSC) was used to assess changes in polymer material including crystallinity; goniometry was used to assess surface hydrophobicity changes; ATR-FTIR was used to assess changes in surface chemistry. The SEM, goniometry and DSC measurements of biotic samples were made for the 180 d (T4) harvest; because the T3 (104 d) biotic measurements were not made for these three assessments of physicochemical changes, these three measures could not be compared with the abiotic (90 d) samples for definitively interpreting evidence of biodegradation owing to the differing time periods (180 d biotic vs 90 d abiotic). Regardless, of these three measures, only the SEM variety statistic (Table 1, main manuscript) indicated differences between abiotic and biotic samples (90 d and 180 d, respectively). Meanwhile, the ATR-FTIR spectral analyses were performed for all biotic time points, and thus the T3 (104 d) biotic samples could be compared for their spectra relative to the close-in-time 90 d abiotic samples to infer differences indicative of biodegradation.

The volatile hydrocarbon content of Brian Seep sediments was determined to establish that the seeps were active and that sediment microbial communities were exposed to hydrocarbons *in situ.* Only methane and ethane were quantifiable (Table S2).

Dissolved oxygen (DO) concentrations of sediment collected from Brian Seep indicated that the sediments were relatively anoxic compared to surrounding seawater (Table S3). These sediment samples had lower salinity, but similar pH, as compared to the seawater (Table S3).

Physical Conditions of the Microcosm Tank System and Abiotic Experiment

As per the Methods, the microcosm tank (Fig. S1) was instrumented with a temperature and light data logger to record water temperature and light intensity (at the depth of the microcosm sediments) every 5 minutes. These data were therefore collected throughout the experiment,

extending from T0 through T4, i.e. from the setup to the last sampling. These data indicate that there were expected diurnal fluctuations in light intensity, i.e. day/night cycles, and that the temperature was likely varying seasonally (although not analyzed in comparison to ambient seawater temperatures *in situ*) (Figs. S3-S4). While it is likely that these fluctuations in light and temperature incurred short, and longer, term (respectively) influences on sediment and coupon-associated bacterial communities, such variations would have been experienced equivalently by all microcosms in the tank, since the seawater was flowing homogenously over each sediment microcosm. Therefore, there were no adjustments to the interpretation of bacterial community data in relationship to such physical data. Further, the multivariate analysis of bacterial communities across all samples indicated that seawater bacterial communities were relatively similar to each other over time, in comparison to time-course sediment and coupon biofilm communities (Fig. 4). This reinforces that light and temperature fluctuations of flowing seawater were not highly influential to the interpretations of bacterial community phylogeny in this experiment.

Further, each sampling event (also as per the Methods) included measuring the influent seawater flowrate into the tank (Table S4), and the DO, pH, and salinity of the seawater and the sediments (Table S3). The additional samples of seawater collected from the marine environment at T0, two days after T4, and on 9/24/21 indicated that the microcosm characteristics were similar to the ambient seawater characteristics (Table S3).

Also as per the Methods, an abiotic simulation (control) of the main microcosm experiment was performed over 90 days in the laboratory to assess if abiotic conditions in the sediments could have contributed to polymer degradation. Since abiotic conditions were of interest, the experiment was similarly instrumented to record water temperature and light intensity. The light intensity was set up to be as close as possible to the average measured during the *ex situ* study (Fig. S3). Similarly, the temperature of the abiotic study was set up to be within the range observed during the *ex situ* study (Fig. S4).

PCR amplification

Amplification, purification, and normalization of samples were performed the same as before (1), in accordance with published dual-index paired-end sequencing methods (2). Briefly, 2.5 μL of extracted DNA (5 ng/μL in minimum) was amplified using the 27F forward primer (5'-AGAGTTTGATCCTGGCTCAG-3') and the 534R reverse primer (5'-

ATTACCGCGGCTGCTGG-3’) (3) which target the V1-V3 region of the 16S rRNA gene with Illumina overhang adapters for 25 cycles (KAPA HiFi HotStart ReadyMix; KAPA Biosystems).

References

1. Li D, Van De Werfhorst LC, Noble RT, Blackwood D, Ervin J, Steets B, Smith J, Holden PA. 2025. Bacterial community analysis of recreational beach waters reveals human fecal contamination and pathogenicity across varying field conditions. Water Res 281:123697.
2. Kozich JJ, Westcott SL, Baxter NT, Highlander SK, Schloss PD. 2013. Development of a dual-index sequencing strategy and curation pipeline for analyzing amplicon sequence data on the MiSeq Illumina sequencing platform. Appl Environ Microbiol 79:5112-5120.
3. Oh J, Byrd AL, Deming C, Conlan S, Kong HH, Segre JA. 2014. Biogeography and individuality shape function in the human skin metagenome. Nature 514:59-64.

Table S1. Characteristics for the plastic and glass substrates (coupons) used in this study, derived from manufacturer data.

Polymer Name	Abbreviation	Composition	M_p (°C)	D_T (°C)	Thickness (µm)	Specific Gravity
Ethylene vinyl alcohol copolymer (Et % 29 mol)	EVOH1	99.7%	150-200	>300	30	1.1-1.24
Ethylene vinyl alcohol copolymer (Et % 38 mol)	EVOH2	99.7%	150-200	>300	30	1.1-1.24
Ethylene vinyl alcohol copolymer (Et % 44 mol)	EVOH3	99.7%	150-200	>300	30	1.1-1.24
Oriented polyethylene terephthalate	PET	>99%	250-265	350-400	12	1.3-1.4
Cellulose (di)acetate	CA	>80%	>100	>250	76.2	1.31
Borosilicate glass	Glass	>99%	1650	NA	130-160	NA

M_p = Melting point; D_T = Decomposition temperature; NA = not provided by the manufacturer and safety data sheet

Table S2. Brian Seep sediment hydrocarbon concentrations from five active bubble plumes sampled on 9/24/21. Standard reporting limits (SRL) for methane and ethane are 0.00010 µg/mL and 0.00019 µg/mL, respectively. Ethylene was not detected (SRL = 0.00022 µg/mL)

	Methane		Ethane	
ID	µg/mL	µmol/L	µg/mL	µmol/L
Brian Seep #1	0.00989	0.6165	0.00023	0.0076
Brian Seep #2	0.01870	1.1656	0.00044	0.0146
Brian Seep #3	0.03537	2.2047	0.00086	0.0286
Brian Seep #4	0.02095	1.3059	0.00070	0.0233
Brian Seep #5	0.00373	0.2325	-	-
average	0.01773	1.1050	0.00056	0.0185
standard deviation	0.01204	0.7505	0.00028	0.0093

Table S3. Physical parameters of the microcosm study setup. DO = dissolved oxygen. Seawater was sampled at three locations: from active bubble plumes at Brian Seep, at the intake screen in the ocean for the seawater system, and from inside the tank containing the microcosms. Sediment was sampled from active bubble plumes at Brian Seep and from the extra set of beakers (no coupons) placed in the seawater tank.

		Seawater				Sediment			
		DO	Salinity	pH	Temperature	DO	Salinity	pH	Temperature
Location	Time point	mg/L	ppt		°C	mg/L	ppt		°C
Brian Seep	12/16/20 (T0)	9.69	32.8	7.44	14.5				
	6/16/21	8.32	32.5	7.60	16.6	0.13	8.88	7.72	17.0
	9/24/21	8.98	32.8	7.22	16.4	0.15	8.53	7.53	15.9
Seawater system intake	6/16/21	8.15	31.7	7.47	16.6				
Seawater tank	12/16/20 (T0)	10.16	33.1	7.40	14.8	4.11			14.5
	12/30/20 (T1)	9.83	31.2	7.41	15.2	1.44			14.1
	1/21/21 (T2)	9.44	32.1	7.33	15.7	1.24			14.6
	3/30/21 (T3)	10.41	33.6	7.44	14.0	1.20			13.3
	6/14/21 (T4)	9.31	32.3	7.33	16.6	0.45			16.5

Table S4. Seawater tank flow rates. At each sampling time point, the flow rate into the tank was measured by recording the time to fill a five-gallon bucket three times.

	Time to fill	Flow	Flow
Time point	seconds	GPM	LPM
12/16/20 (T0)	74.55	4.0	15.2
	74.66	4.0	15.2
	75.21	4.0	15.1
12/30/20 (T1)	85.19	3.5	13.3
	84.84	3.5	13.4
	84.90	3.5	13.4
1/21/21 (T2)	89.98	3.3	12.6
	89.62	3.3	12.7
	89.67	3.3	12.7
3/30/21 (T3)	98.00	3.1	11.6
	99.45	3.0	11.4
	99.05	3.0	11.5
6/14/21 (T4)	132.33	2.3	8.6
	127.59	2.4	8.9
	128.86	2.3	8.8
Average		3.2	12.3

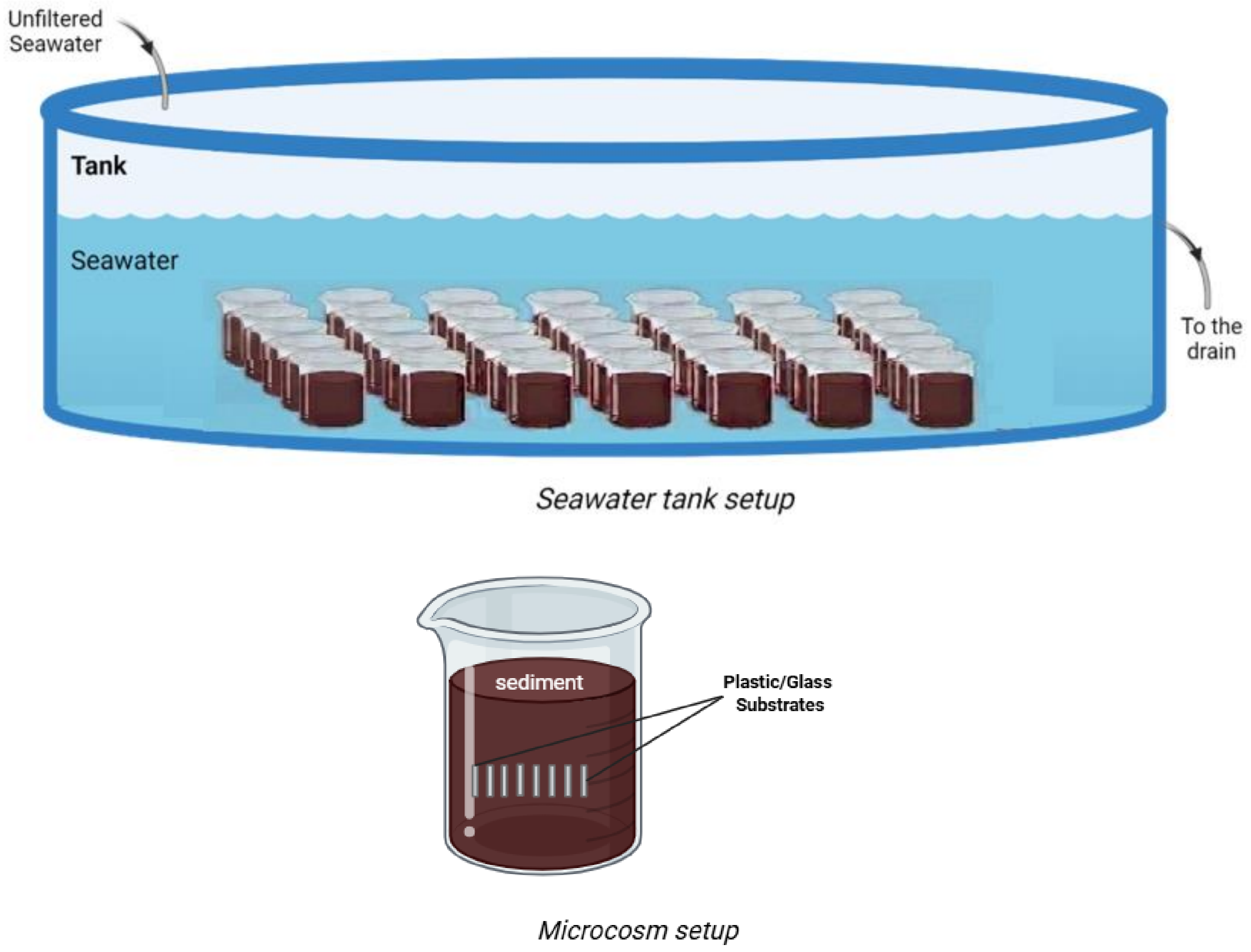

Figure S1. The microcosm and seawater tank were set up for this study in the Marine Operations Facility at UC Santa Barbara. Illustration generated on Biorender.

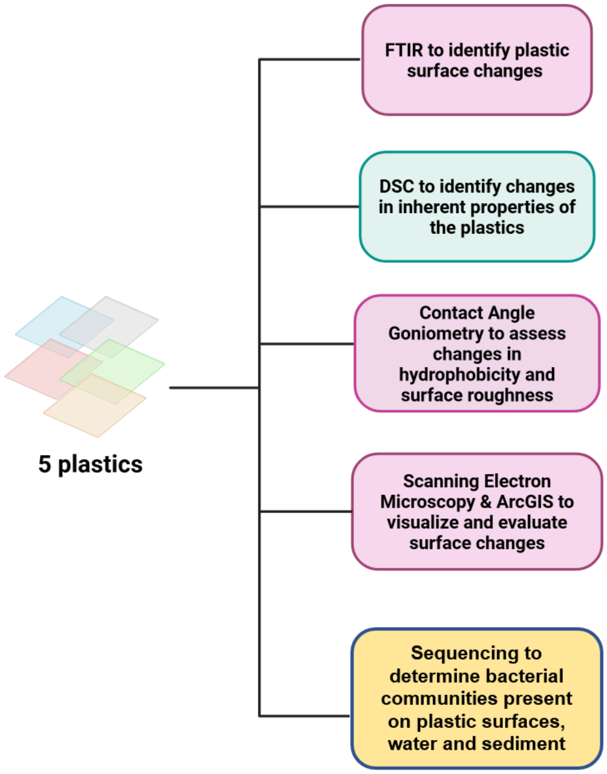

Figure S2. The analysis pipeline for the plastic substrates (coupons) harvested over time from microcosms in this study.

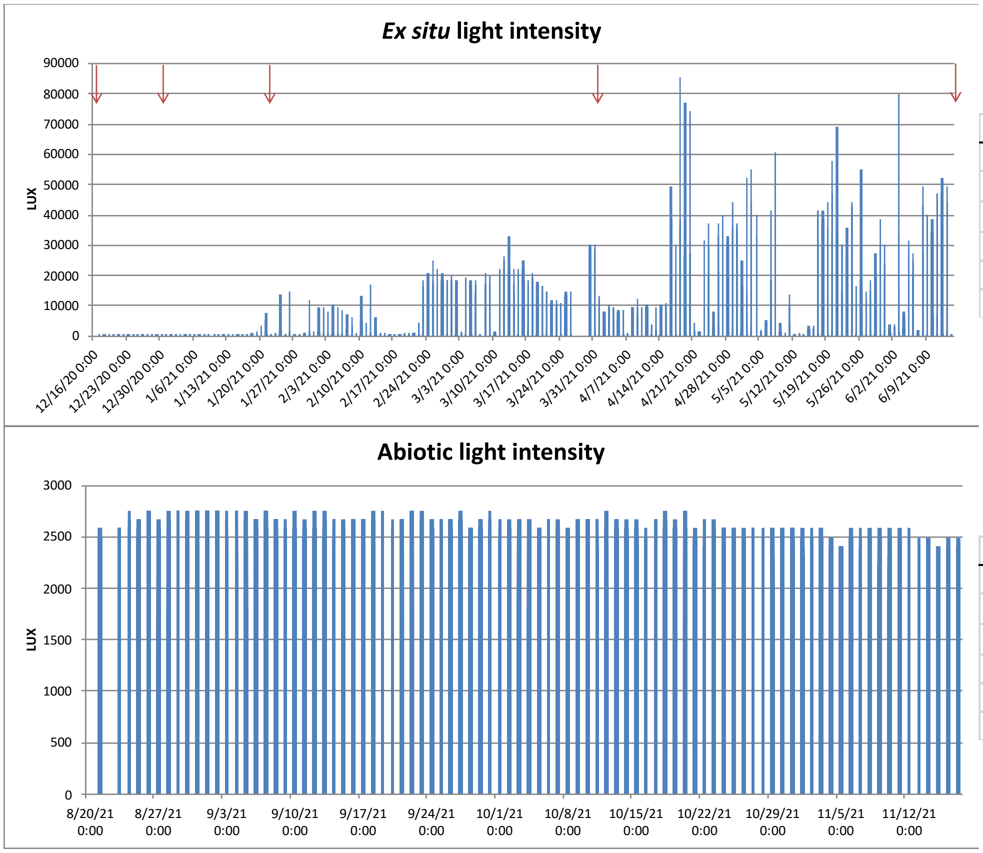

Ex situ	
255.7	avg LUX
0	min LUX
85422.8	max LUX
0	median
50791	measurements

Abiotic	
346.9	avg LUX
0	min LUX
2755.6	max LUX
0	median
25676	measurements

Figure S3. HOBO light measurement results from the *ex situ* and abiotic microcosm studies. Red arrows indicate the approximate sampling time points for the *ex situ* study (T0, T1, T2, T3, T4). Lighting conditions for the abiotic study were set to be as close as possible to the average amount of light measured during the *ex situ* study. The righthand tables in each panel provide the mean (avg LUX) and median, as well as the minimum (min LUX) and maximum (max LUX) light intensities occurring over the total number of measurements (bottom row of each table).

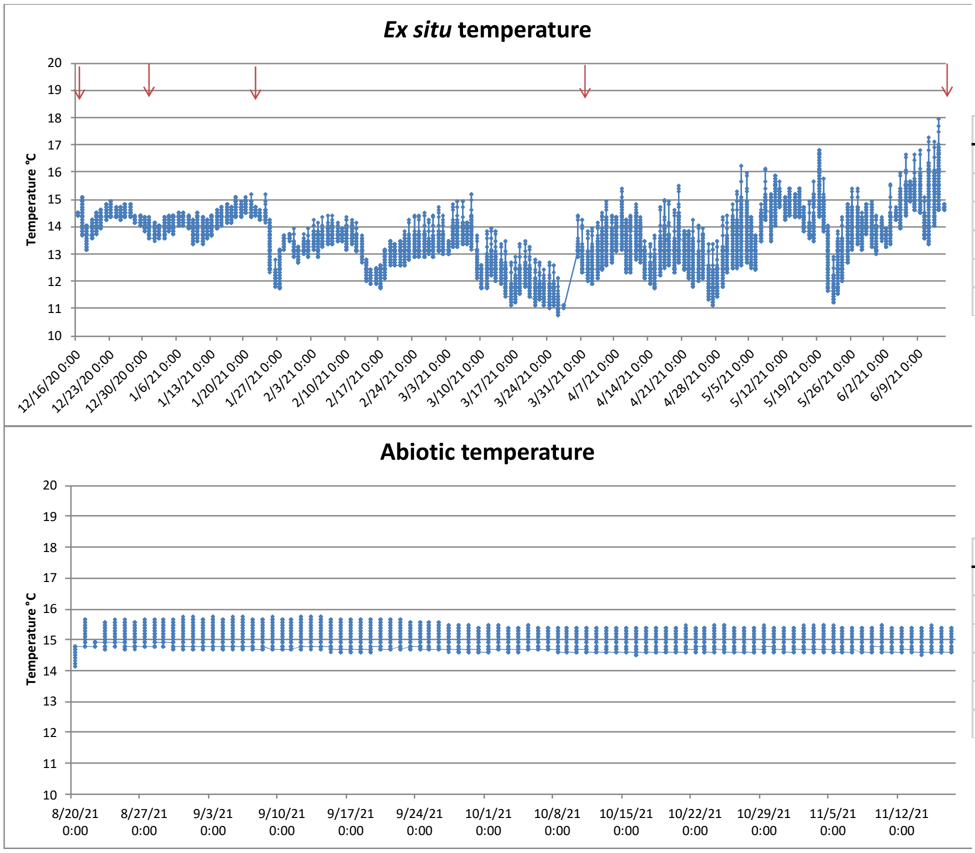

Ex situ	
13.475	average °C
10.748	minimum °C
17.950	maximum °C
13.558	median °C
50791	measurements

Abiotic	
14.845	average °C
14.134	minimum °C
15.760	maximum °C
14.804	median °C
25676	measurements

Figure S4. HOBO temperature measurement results from the *ex situ* and abiotic microcosm studies. Red arrows indicate the approximate sampling time points for the *ex situ* study (T0, T1, T2, T3, T4). The temperature for the abiotic study was set to be within range of that measured in the *ex situ* study. The righthand tables in each panel provide the mean (average) and median, as well as the minimum and maximum, temperatures (all °C) occurring over the total number of measurements (bottom row of each table).

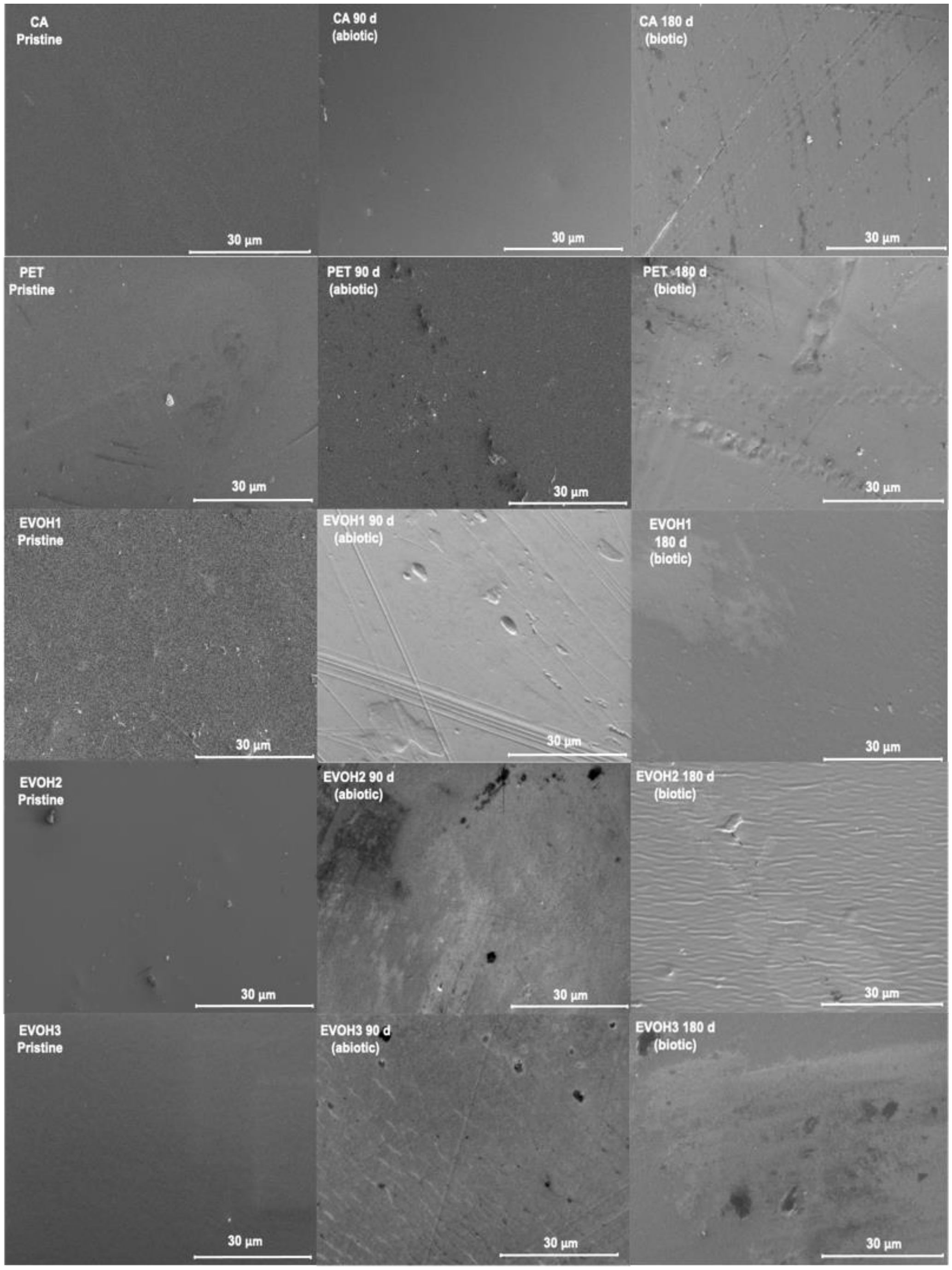

Figure S5. Representative SEM micrographs of the polymer substrate surfaces, specifically pristine, abiotic (90 d), and biotic (T4). All images were captured at a magnification of 2600×. Glass control substrates were not included in the analysis, as changes were not observed.

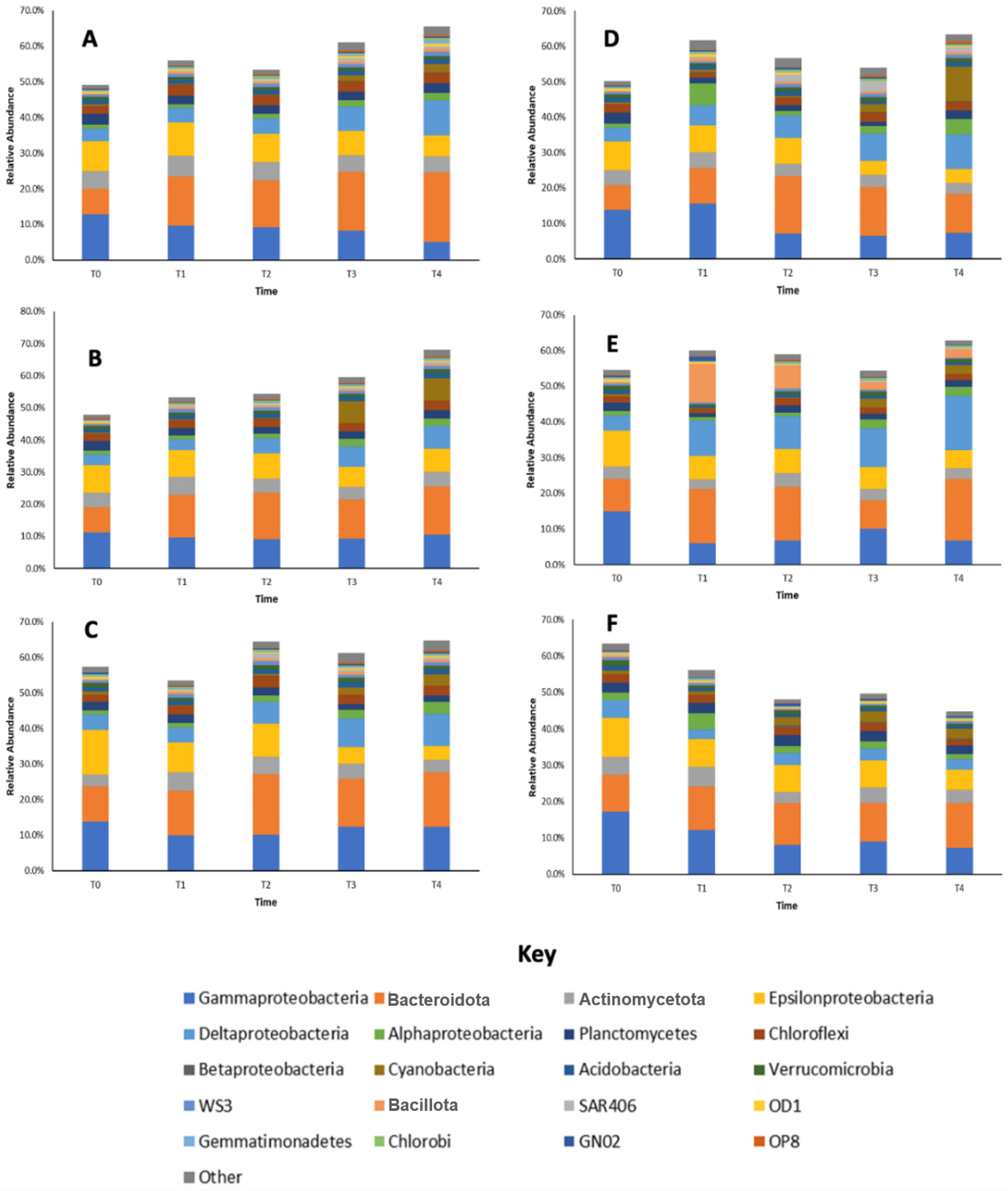

Figure S6. Relative abundances of the 20 most abundant bacterial phyla and superclasses on all substrates over time (A = EVOH1, B = EVOH2, C = EVOH3, D = PET, E = CA, F = Glass Control).

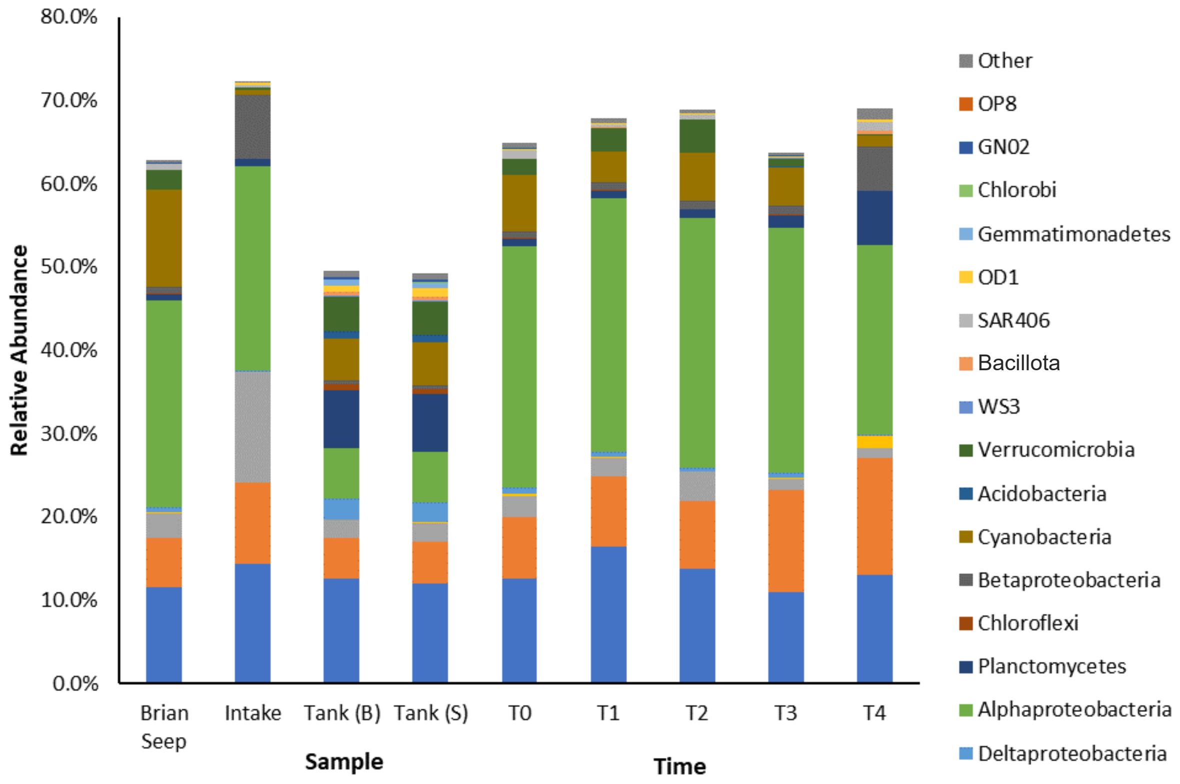

Figure S7. Average relative abundances of the 20 most abundant bacterial phyla and superclasses in the tank seawater over time (T0 to T4) and the seawater collected from Brian Seep and the seawater system intake two days after T4, and the tank surfaces (B = bottom, S = side) for comparison.

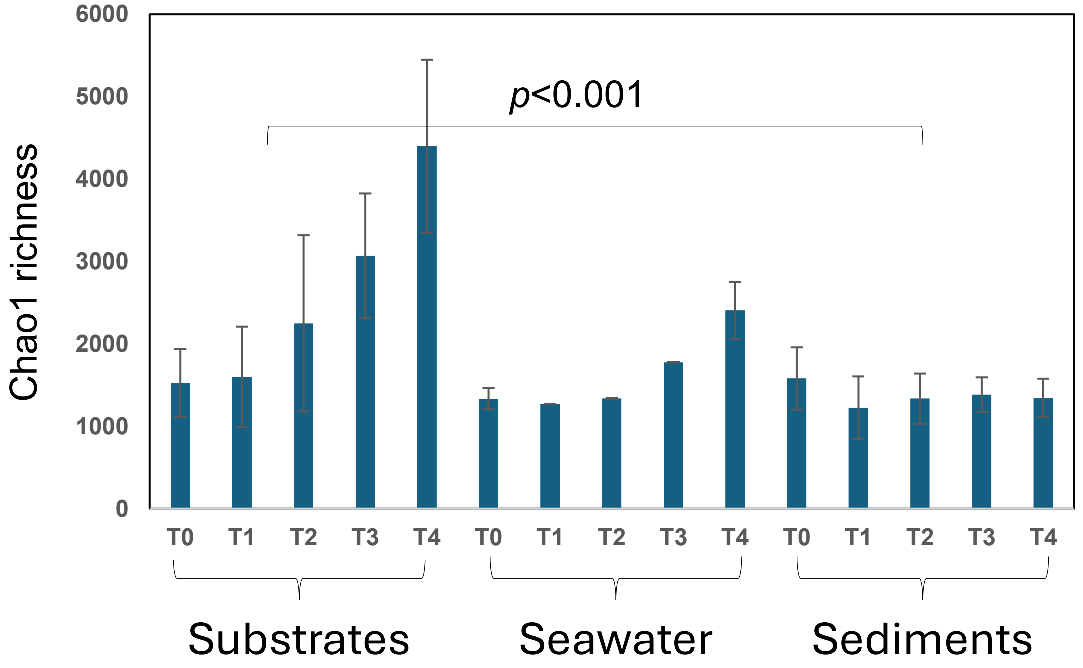

Figure S8. The average and standard deviation of Chao1 richness values of bacterial communities on all substrates (n=90), in seawater (n=9) and sediments (n=31) of this study. The detailed Chao1 richness values are shown in Table S6. The *p* value in the figure shows the significant difference between substrates and sediments.

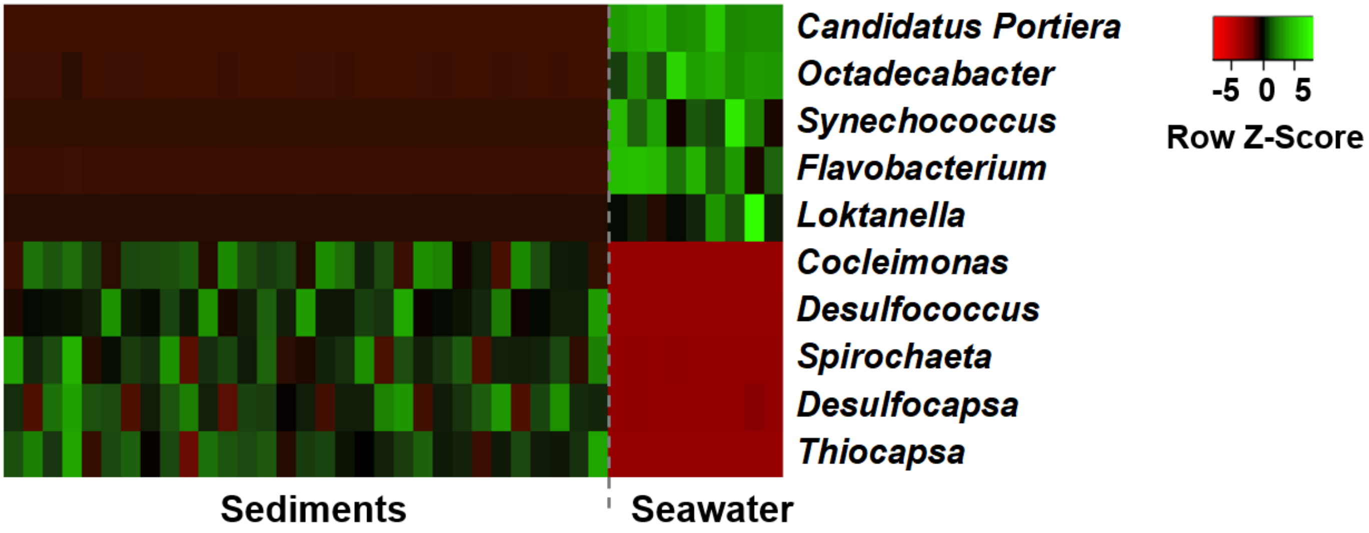

Figure S9. A heatmap of relative abundances of bacterial genera specifically associated with sediments (n=31) and seawater (n=9). The top 5 bacterial genera with the highest average relative abundance were selected and shown in the order from top to bottom. From left to right, the first sediment sample was the homogenized sediments used to fill the microcosm before incubation, followed by the substrate-containing sediments collected from the microcosm in the order of EVOH1 (n=5), EVOH2 (n=5), EVOH3 (n=5), PET (n=5), cellulose acetate (n=5), and glass (n=5). For each microcosm, the sediment samples are in the order of sampling times i.e., T0 to T4. The seawater samples included samples individually taken from incubation tank. From left to right, each column corresponds with each sampling time (T0 to T4), sample from Brian Seep at T0 and two days after T4, as well as one sample from the seawater system intake location two days after T4. The bacterial genera were shown as the relative abundances (normalized by Z-score across all data sets).
